# Korean Temple Food as Plant-Based Food Culture: A Social Big Data Analysis of Changing Online Food Discourse Across the COVID-19 Transition

**DOI:** 10.3390/foods15193390

**Published:** 2026-09-23

**Authors:** Changkyun Kang, Jangheon Han

**Affiliations:** 1 College of Humanitas, Kyung Hee University, 26 Kyungheedae-ro, Dongdaemun-gu, Seoul 02447, Republic of Korea; kazagi@khu.ac.kr; 2 Department of Tourism Administration, Yong In University, Yongin-si 17092, Gyeonggi-do, Republic of Korea

**Keywords:** Korean temple food, plant-based food culture, online food discourse, health-oriented eating, social big data, semantic network analysis, COVID-19 transition

## Abstract

This study examines how Korean-language online discourse surrounding Korean temple food evolved across the COVID-19 transition, with particular attention to its changing meanings as a traditional plant-based food culture. A cleaned corpus of 24,353 online documents was analyzed across three three-year periods: pre-COVID-19 (1 January 2017–31 December 2019), COVID-19 (1 January 2020–31 December 2022), and post-endemic (1 June 2023–31 May 2026). Sixty core keywords were selected for each period and examined using frequency analysis, normalized weighted strength (NWS), degree and closeness centrality, semantic network analysis, CONCOR, and Jaccard similarity. Before COVID-19, discourse centered on vegetarianism, cooking, ingredients, dining, and traditional Buddhist food culture. During the pandemic, veganism, nature, health, and healing became more prominent, strengthening associations with plant-based and health-oriented eating. In the post-endemic period, this food identity remained visible while experiential, wellness-related, and media-driven meanings became more distinct. Jaccard similarity was 0.739 for the pre-COVID-19 versus COVID-19 comparison, 0.600 for COVID-19 versus post-endemic, and 0.519 for pre-COVID-19 versus post-endemic, indicating substantial continuity alongside greater compositional differentiation across more temporally distant comparisons and supporting an interpretation of gradual reconfiguration rather than abrupt change. Overall, Korean temple food has been recontextualized as a broader plant-based food culture in which its traditional Buddhist culinary foundations coexist with health, wellness, experiential consumption, and cultural meanings within contemporary online food discourse.

## 1. Introduction

Food serves not only as a source of physiological nourishment but also as a cultural medium through which social relationships, lifestyles, and identities are expressed [1,2]. Traditional foods, in particular, embody local history, collective memory, and culturally embedded knowledge, allowing dietary practices to retain social and cultural significance as they adapt to changing patterns of consumption [3]. In contemporary food research, this perspective has become increasingly relevant as consumers show growing interest in plant-based eating, health-oriented diets, ethical consumption, and food practices grounded in naturalness and tradition. Korean temple food provides a distinctive context in which these dimensions converge. Rooted in Buddhist dietary practice, it combines plant-based ingredients, culturally specific preparation principles, and a philosophy of moderation, while increasingly being encountered beyond monastic settings as part of contemporary food consumption and lifestyle discourse.

Korean temple food is grounded in Buddhist principles of respect for life, moderation, purity, and spiritual practice [4]. Unlike conventional vegetarian diets, it excludes meat and fish while also restricting the use of the five pungent vegetables traditionally considered disruptive to Buddhist practice. Its reliance on seasonal ingredients and naturally derived plant foods further distinguishes it as a culturally specific form of Buddhist cuisine [5]. Temple food can therefore be understood through the interconnected principles of seasonality, locality, moderate consumption, and plant-based eating. Previous research has identified a range of motivations associated with temple food consumption, including health orientation, ethical vegetarianism, meditative mindfulness, educational experience, taste, and environmental concern [6]. These characteristics suggest that temple food should not be viewed solely as religious or traditional cuisine. Rather, it represents a distinctive plant-based food culture in which Buddhist values, natural ingredients, health-oriented eating, and contemporary wellness-related meanings intersect.

The contemporary relevance of temple food has also expanded through a range of food-service and experiential consumption settings. Specialized temple food restaurants, cooking classes, Templestay programs, and traditional cultural activities have enabled temple food to reach consumers outside monastic practice. More broadly, food and gastronomic experiences have been shown to contribute to cultural engagement, satisfaction, place-related meanings, and memorable consumption experiences [7,8,9]. Within the context of Korean temple food, these settings embed plant-based ingredients and traditional preparation practices within broader experiences involving culinary learning, health, wellness, and cultural interpretation [10,11]. Research on gastronomic experience and co-creation further suggests that participation in food-related activities can generate experiential value extending beyond the act of eating itself [12]. Temple food has consequently been approached not only as a traditional cuisine but also as a food experience associated with physical health, psychological restoration, cultural learning, and wellness [10]. This broader positioning highlights the increasing relevance of temple food to contemporary consumers seeking foods that combine naturalness, healthfulness, cultural authenticity, and experiential value.

The COVID-19 pandemic provided an important social context in which these meanings could be further reconfigured. During this period, food-related behaviors, sustainable food consumption, health-oriented dietary concerns, and plant-based dietary practices also changed substantially [13,14,15]. At the same time, Korean temple food continued to undergo popularization and globalization. Previous research has discussed its development in relation to the preservation and reinterpretation of tradition, sustainable food culture, and the broader globalization of Korean cuisine [5,16]. Buddhist nuns and recognized temple food practitioners have played an important role in recovering, transmitting, and internationalizing temple food traditions, while contemporary representations have increasingly connected temple food with ethical living, ecological concerns, and changing food lifestyles [5,16]. More recently, temple food has also entered popular and commercial food culture and has been examined through the perspective of experiential co-creation within plant-based food consumption [17]. These developments indicate that the contemporary meaning of temple food is shaped by the coexistence of traditional Buddhist food principles and emerging food-related concerns involving health, plant-based eating, wellness, and mediated cultural consumption.

Existing research on Korean temple food has examined consumer perceptions, preferences, popularization, satisfaction, global market segmentation, gastronomic experiences, and wellness-related applications [18,19,20,21]. Studies involving domestic and international patrons of temple food restaurants have linked temple food with vegetarian eating, mild flavors, dieting, healthfulness, and natural ingredients. Research involving Templestay participants and temple food consumers has likewise examined satisfaction, recommendation and revisit intentions, while other studies have explored the potential of temple food as a Korean culinary resource capable of combining health-oriented qualities with cultural storytelling. International research, although more limited in volume than the Korean literature, has similarly interpreted temple food beyond the narrow category of religious cuisine, connecting it with sustainable plant-based food, gastronomic experience, wellness consumption, cultural heritage, and the globalization of Korean food [5,6,11,12,17]. Collectively, these studies demonstrate that temple food can be understood simultaneously as a Buddhist food tradition, a form of plant-based consumption, a health- and wellness-related food experience, and a culturally embedded form of contemporary food consumption.

Nevertheless, important gaps remain. Existing temple food research has largely examined consumer perceptions, satisfaction, values, behavioral intentions, and food experiences through cross-sectional surveys of restaurant patrons, Templestay participants, or other specific consumer groups. These studies provide important evidence on individual-level responses but offer more limited insight into how food-related meanings are collectively formed and reconfigured within the broader digital information environment. This distinction is relevant to food-consumer research because online discourse provides a context in which attributes such as plant-based eating, health, naturalness, cultural authenticity, and experiential value are socially articulated and connected, even though such discourse does not directly measure individual consumer behavior. A longitudinal analysis of online discourse can therefore complement consumer-based research by identifying how the semantic context surrounding Korean temple food changes over time.

Against this background, the present study uses social big data to examine changes in Korean-language online discourse surrounding Korean temple food across the pre-COVID-19, COVID-19, and post-endemic periods. Frequency analysis is used to identify changes in keyword prominence, while normalized weighted strength, degree centrality, and closeness centrality examine relational intensity, direct connectivity, and network accessibility. CONCOR analysis identifies period-specific semantic clusters, and Jaccard similarity assesses continuity and change in core keyword composition. By integrating these analytical layers, the study examines how traditional Buddhist culinary and plant-based meanings persisted while health-, wellness-, experiential-, place-based-, and media-related meanings became differently configured over time.

The study contributes to food and consumer research by conceptualizing Korean temple food as a culturally embedded plant-based food culture and by demonstrating how traditional food meanings coexist with contemporary consumer-relevant meanings within digital discourse. It also provides a longitudinal framework for examining how culturally specific food practices are socially recontextualized within changing consumption and information environments.

## 2. Literature Review

### 2.1. Korean Temple Food as Buddhist Plant-Based Food Culture

Korean temple food refers to the food traditionally prepared and consumed by monks and practitioners in Buddhist temples and reflects Buddhist principles such as respect for life, moderation, purity, gratitude, and spiritual discipline [22]. Similar to vegetarian cuisine more broadly, it excludes animal-based ingredients such as meat and fish, but its distinctiveness extends beyond these dietary restrictions. The use of the five pungent vegetables is also restricted because they are traditionally regarded as disruptive to spiritual practice. Temple food is therefore understood not simply as nourishment but as part of a disciplined way of life oriented toward spiritual cultivation, wisdom, and self-reflection. Although rooted in Buddhist monastic practice, its contemporary meaning has increasingly expanded to encompass plant-based eating, health orientation, ethical consumption, and sustainable food practices.

This distinctive character is also evident in its ingredients and methods of preparation. Korean temple food relies heavily on naturally derived plant ingredients, including wild greens, field vegetables, roots, fruits, seaweed, and grains [4,23]. Seasonings and artificial flavor enhancers are generally used sparingly so that the inherent taste and aroma of the primary ingredients can be preserved. Eating itself is embedded in Buddhist practice, giving temple food religious and cultural significance extending beyond culinary technique [24]. Park et al. [6] characterize Korean temple food as a plant-based dietary practice distinguished by the avoidance of meat, the use of seasonal and local ingredients, and moderate consumption. They further identify consumption motives including health orientation, ethical vegetarianism, meditative mindfulness, educational experience, taste, and environmental concern. These characteristics position temple food not only as food associated with religious practice but also as a contemporary plant-based food culture linked to naturalness, vegetarianism, health, wellness, and sustainability.

Temple food also constitutes a culturally embedded form of dietary practice in which Korean culinary traditions and Buddhist culture converge. Bae [25] locates the heritage value of Korean temple food and Balwoo Gongyang in Buddhist precepts, ecological wisdom, modest everyday foodways, communal sharing, frugality, and practices intended to minimize waste. From this perspective, temple food is more than a collection of recipes or individual dishes; it represents a food culture in which Buddhist values are embedded in culinary knowledge, ingredient selection, preparation, and everyday eating practices. Contemporary temple food, however, is increasingly consumed outside monastic settings through restaurants, culinary education, cultural programs, and other experiential settings. Jeon and Ko [11] examined this broader development through consumption value, food satisfaction, happiness, and behavioral intention among international consumers of Korean temple food. Moon and Lee [18] similarly found that Korean and international restaurant patrons associated temple food with vegetarian eating, mild taste, dieting, and healthfulness. Collectively, these findings indicate that temple food is increasingly perceived not only through its religious and cultural origins but also as a natural and health-oriented form of plant-based food consumption accessible to broader consumer groups.

Accordingly, this study conceptualizes Korean temple food as “Buddhist plant-based food culture.” This conceptualization moves beyond treating temple food solely as either vegetarian food or traditional cuisine. Instead, it provides a theoretical basis for understanding temple food as a multidimensional dietary culture in which Buddhist respect for life, natural ingredients, seasonality, moderation, health orientation, sustainability, and wellness-related values intersect. Such a perspective is particularly relevant when examining changes across the COVID-19 transition, during which public attention to health, natural foods, vegetarianism, veganism, and sustainable eating became increasingly salient.

### 2.2. Temple Food as Health-Oriented and Experiential Food Consumption

The contemporary development of Korean temple food is increasingly evident in health-oriented and experiential forms of food consumption. Once encountered primarily within Buddhist temples and monastic practice, temple food is now available to broader consumer groups through specialized restaurants, cooking classes, Templestay programs, and traditional cultural activities. These settings allow consumers to experience temple food not simply as a meal but as a combination of plant-based eating, culinary learning, cultural interpretation, health orientation, and experiential consumption. Son and Xu [26] showed that Buddhist temple food could contribute to experiences involving novelty, sensory pleasure, authenticity, and cultural exploration among Western visitors. Kim et al. [27] similarly identified Buddhist temple cuisine as a potential healthy alternative food choice as well as a specialized form of food experience. These studies suggest that the contemporary value of temple food derives from both its distinctive dietary characteristics and the experiential contexts in which it is consumed.

Research on temple food consumption has also examined the relationships among food-related attributes, satisfaction, and subsequent behavioral responses. Park and Chong [28] compared Buddhist and non-Buddhist Templestay participants and found that perceptions of temple food were associated with satisfaction and recommendation intention. Shin et al. [29] likewise reported relationships among involvement with temple food, satisfaction with the experience, recommendation intention, and revisit intention. Although these studies were conducted within Templestay settings, their findings demonstrate that temple food can function as an identifiable component of a broader food experience rather than merely as an incidental meal.

Its potential as a restaurant and educational food product has also received attention. Kim and Hong [30] examined temple food choice attributes, satisfaction, and repurchase intention among participants in temple food courses. Attributes concerning perceived nutritional value, taste and aroma, natural ingredients, and health-related functions were associated with consumer evaluations, while natural ingredients and nutritional considerations were also related to repurchase intention. These findings should be interpreted as consumer evaluations of temple food attributes rather than direct evidence of clinical or nutritional effects. Nevertheless, they indicate that naturalness, perceived healthfulness, sensory characteristics, and food quality constitute important dimensions through which consumers evaluate temple food.

Temple food can therefore be interpreted through the combined perspectives of health-oriented food choice and experiential consumption. Kim et al. [27] examined perceptions, attitudes, and subjective norms toward Buddhist temple cuisine and proposed its potential as a healthy alternative food choice. Park and Cho [31], using the experience economy framework, examined entertainment, education, esthetics, and escapism and found these experiential dimensions to be positively associated with perceived value, satisfaction, and behavioral intention. Their generational comparison further indicated that consumers may differ in how they evaluate temple food experiences. Taken together, these studies suggest that the value of temple food is formed not only through its ingredients or religious background but also through the way consumers experience, interpret, and assign meaning to its culinary and cultural attributes.

More recent studies have further connected temple food with wellness and subjective well-being. Jang [32] examined physical and mental wellness, life satisfaction, subjective happiness, and quality of life among consumers who regarded temple food as healing food. Physical and mental wellness were positively related to subjective happiness, while mental wellness was also associated with life satisfaction. These findings suggest that consumers may attach broader wellness-related meanings to temple food beyond its role as a healthy dietary option. Jeong and Lee [33] further investigated the relationships among consumers’ need for uniqueness, memorable temple food experiences, subjective well-being, and behavioral intentions, while considering the moderating role of health consciousness. Their findings indicate that temple food can provide differentiated experiential and emotional value and that its healthy and vegetarian-oriented image may be particularly relevant to health-conscious consumers.

This broader experiential dimension does not diminish the food-centered identity of temple cuisine. Rather, it suggests that temple food consumption occurs at the intersection of dietary characteristics and culturally situated experiences. Healthfulness, naturalness, plant-based ingredients, cultural authenticity, and memorable food experiences can jointly shape how consumers interpret and evaluate temple food [34,35]. In this sense, tourism and Templestay represent contextual settings through which temple food is encountered rather than the primary conceptual endpoint of the food itself.

Overall, Korean temple food now extends beyond the conventional categories of Buddhist monastic cuisine and traditional food. Its contemporary meaning incorporates plant-based eating, health-oriented food choice, gastronomy, wellness-related consumption, and culturally embedded food experiences. Nevertheless, much of the existing literature has relied on survey-based research involving specific groups, including Templestay participants, cooking-class attendees, temple food restaurant customers, and international visitors. Consequently, limited attention has been given to how temple food is represented and interpreted within broader online public discourse and how this meaning structure has evolved over time.

### 2.3. Social Big Data, COVID-19 Transition, and the Recontextualization of Temple Food Discourse

Social big data and text-mining approaches provide an important means of examining how food-related meanings are produced, circulated, and reorganized in digital environments. Across tourism, hospitality, restaurant, and food research, online narratives, reviews, social media posts, and other forms of digital content have been used to examine consumer perceptions, experiences, and food-related discourse [36,37,38]. Unlike conventional surveys, which typically collect responses within researcher-defined categories, social big data draws on large volumes of spontaneously generated and unstructured online text. Such data can therefore capture publicly expressed interests, experiences, evaluations, and semantic relationships that may not be fully represented by predefined survey measures [39,40,41,42]. Text mining, semantic analysis, and related computational approaches are particularly useful for identifying recurring themes and relational structures within large-scale textual corpora [43,44].

Recent food research has further demonstrated the value of social media and online big data for examining changes in food-related meanings and discourse. Jang et al. [45], for example, used social media big data to compare food delivery discourse before and after the COVID-19 outbreak, combining text mining and CONCOR analysis to identify temporal differences in food-related perceptions and semantic structures. Pilař et al. [46] analyzed large-scale food-related Twitter data and identified distinct online communities associated with healthy, homemade, vegan, and other food-related themes. More recently, Jung et al. [47] applied social media big data and text-mining approaches to examine public perceptions of food insecurity and their temporal characteristics. Together, these studies demonstrate that digitally generated food discourse can be used to examine not only the prominence of food-related topics but also changes in their relational and temporal organization.

The relevance of these methods is particularly evident for food topics that are simultaneously shaped by consumer experience, cultural representation, media exposure, and institutional communication. Online food discourse is not produced exclusively by individual consumers; it may also include restaurants, culinary professionals, organizations, media outlets, and other actors involved in producing and circulating food-related meanings. Accordingly, social big data should not automatically be interpreted as a statistically representative measure of individual consumer attitudes. Rather, it can be used to examine the broader semantic environment in which particular foods are discussed and socially contextualized. This distinction is especially important for Korean temple food, whose online representation spans individual dining experiences, recipes, restaurants, Buddhist cultural practices, health and wellness information, Templestay, media content, and K-food discourse.

Despite growing use of digital textual data, temple food research has remained largely centered on consumer perceptions, values, satisfaction, and behavioral intentions, with comparatively little attention paid to long-term shifts in public discourse or semantic structure. Reviews of Korean temple food research likewise indicate that quantitative and survey-based approaches have remained prominent, while recurring research themes have focused on recognition, value, satisfaction, and intention [22]. Consequently, existing studies provide relatively limited evidence regarding how temple food has been socially reinterpreted over time as plant-based eating, health-conscious consumption, veganism, wellness, and other food-related values have gained visibility.

The COVID-19 pandemic provides a particularly meaningful context for examining these temporal changes. Although the pandemic disrupted tourism and mobility and prompted broader reconsideration of established practices [48], it also generated substantial changes in food-related behavior and information seeking. Greater attention was directed toward food security, dietary practices, nutrition-related information, home-based living, cooking, and recipes [49]. Public interest in food sustainability, sustainable diet management, healthy eating, and flexitarian consumption also changed during this period [50]. Vegan consumption became more closely associated with concerns regarding health, food safety, animal welfare, environmental issues, and ethical consumption [51]. These developments suggest that COVID-19 should be understood not only as a tourism crisis but also as a period in which the social meanings attached to food, dietary practices, and plant-based consumption were actively reconsidered.

Previous longitudinal social big data studies provide a methodological basis for examining such changes across distinct periods. Lee and Jang [52], for example, divided online perceptions into pre-COVID-19, COVID-19, and post-COVID-19 stages and examined differences using social media big data. Youn and Kim [53] similarly analyzed temporal changes through TEXTOM, TF-IDF, CONCOR, and related network techniques. Although these studies addressed tourism- or nature-related contexts, their period-comparative designs demonstrate the value of treating COVID-19 as a sequence of changing discourse environments rather than reducing temporal change to a simple before-and-after distinction.

This temporal perspective is particularly relevant to Korean temple food because its core characteristics intersect with several food-related concerns that became increasingly visible during and after the pandemic. Before COVID-19, online discourse may have been more strongly connected with traditional Buddhist cuisine, specialized temple food restaurants, cooking practices, ingredients, religious food practices, and established cultural experiences [26]. During the pandemic, meanings associated with health, natural foods, vegetarianism, veganism, cooking, recipes, and healing may have become more prominent, consistent with broader changes in health-conscious and plant-based food consumption [49,50,51]. In the post-endemic period, the underlying plant-based and traditional food identity of temple food may have persisted while becoming increasingly connected with wellness, experience, online reviews, media exposure, prominent culinary practitioners, and renewed mobility. Earlier research on the reinvention and globalization of Korean temple food [24], together with more recent studies of Buddhist nuns, culinary practitioners, and plant-based food consumption [5,17], provides a basis for interpreting such changes as recontextualization rather than replacement of tradition.

Accordingly, the division of the analysis into pre-COVID-19, COVID-19, and post-endemic periods serves a purpose beyond chronological convenience. The three-period design provides an analytical framework for tracing how Korean temple food discourse may have developed from a structure centered predominantly on traditional Buddhist cuisine and culinary practices toward a broader plant-based food culture incorporating health-oriented eating, veganism, wellness-related meanings, experiential consumption, and digitally mediated food discourse. The temporal comparison therefore makes it possible to examine not only whether individual keywords increased or decreased in prominence but also how the broader semantic configuration surrounding Korean temple food changed across a major period of social and dietary transformation.

## 3. Materials and Methods

### 3.1. Research Questions and Analytical Framework

This study adopts an exploratory comparative research design to examine changes in the structure and meaning of online discourse surrounding Korean temple food across the COVID-19 transition. The analysis is organized into three periods: pre-COVID-19, the COVID-19 period, and the post-endemic period. This temporal framework is designed to trace how temple food retained its identity as a traditional Buddhist plant-based food culture while becoming increasingly associated with health-oriented eating, veganism, wellness-related meanings, experiential food consumption, and digitally mediated food discourse. Whereas much of the existing literature has focused on individual-level attitudes and behaviors, including consumer perceptions, satisfaction, perceived value, and behavioral intentions, the present study shifts attention to the broader structure of socially produced online discourse and its temporal reconfiguration.

To address this objective, the study first compares the major keywords and their composition across the three periods and then examines the semantic relationships among these keywords. Centrality analysis is used to identify keywords occupying structurally prominent positions within the temple food discourse. CONCOR analysis is employed to identify semantic subgroups characterizing each period, while Jaccard similarity is used to quantify continuity and change in the composition of the core keyword sets across periods. By integrating these analytical results, the study examines which meanings persisted, which became newly salient or declined in prominence, and how the semantic context surrounding Korean temple food was progressively reconfigured. Particular attention is given to the extent to which a discourse rooted in traditional Buddhist cuisine developed into a broader plant-based food culture incorporating health-oriented eating, veganism, wellness-related meanings, experiential consumption, and mediated food discourse. Based on this analytical framework, the following research questions are proposed:

**RQ1**.  *How do the core keywords and their composition differ across the pre-COVID-19, COVID-19, and post-endemic periods in online discourse on Korean temple food?*

**RQ2**.  *How do the structural positions of temple food-related keywords and their CONCOR cluster structures differ across the three periods?*

**RQ3**.  *To what extent do the core keyword sets show continuity and change across the three periods, as measured by Jaccard similarity?*

**RQ4**.  *What overarching pattern of continuity and semantic reconfiguration emerges when the keyword, network, cluster, and similarity results are considered together across the COVID-19 transition?*

### 3.2. Analysis Period and Data Collection

This study divided the observation period into three phases to examine temporal changes in online discourse surrounding Korean temple food across the COVID-19 transition: pre-COVID-19, the COVID-19 period, and the post-endemic period. Each analytical window covered three years to ensure temporal comparability. The pre-COVID-19 period extended from 1 January 2017 to 31 December 2019, and the COVID-19 period from 1 January 2020 to 31 December 2022. The post-endemic period was operationally defined as 1 June 2023 to 31 May 2026. The starting point of this period was anchored to the official downgrade of Korea’s COVID-19 crisis alert level from “Serious” to “Warning,” which took effect on 1 June 2023 [54]. Accordingly, the term post-endemic in this study refers to a policy and social transition toward sustained COVID-19 management rather than the epidemiological disappearance of the disease. January through May 2023 was excluded as a transitional interval between the acute pandemic-response phase and the revised domestic management regime. The final observation cutoff was 31 May 2026; no documents dated after this point were included in the analytical corpus. Thus, the three periods represent comparable temporal windows covering pre-pandemic conditions, the pandemic period, and the subsequent transition toward normalized social activities, mobility, and consumption practices.

Online unstructured texts were collected using TEXTOM, a web-based text collection and analysis platform, with “사찰음식” (Korean temple food) as the primary search term. Data were obtained from Naver Blog, Naver Café, Naver Knowledge iN, Daum Blog, Daum Café, and Facebook-related web documents indexed through Google. The same search term and source configuration were applied across all three analytical periods to maintain comparability. Because data retrieval was centered on the Korean search term “사찰음식” and primarily Korean online platforms, the findings are interpreted as patterns of Korean-language online discourse rather than as evidence of global discourse on temple food. The use of a single focal search term improved temporal consistency across periods but may have excluded relevant discussions expressed through alternative terms or related concepts. The collected materials included individual accounts of food consumption and experiences as well as content produced by Buddhist temples and organizations, restaurants, cultural and educational institutions, tourism-related organizations, and media outlets. Accordingly, the dataset was not treated as a statistically representative sample of a specific consumer population. Rather, it was conceptualized as a corpus of socially produced online discourse through which meanings surrounding Korean temple food were generated and circulated. This study did not involve surveys, interviews, experiments, participant recruitment, or direct interaction with individual users; it analyzed retrospectively collected online textual materials.

The initial pre-COVID-19 dataset comprised 8880 documents, including 2865 from Naver Blog, 2098 from Naver Café, 2944 from Naver Knowledge iN, 341 from Daum Blog, 426 from Daum Café, and 206 from Google-indexed Facebook-related web documents. During the COVID-19 period, 8633 documents were collected from the same sources, comprising 2909, 1813, 2922, 354, 427, and 208 documents, respectively. The post-endemic dataset contained 10,535 documents: 3693 from Naver Blog, 2483 from Naver Café, 3006 from Naver Knowledge iN, 534 from Daum Blog, 571 from Daum Café, and 248 from Google-indexed Facebook-related sources. Across the three periods, the initial corpus therefore consisted of 28,048 documents.

All collected documents were subjected to the same preprocessing procedure across periods. After cleaning, 7823 of 8880 documents were retained for the pre-COVID-19 period, 7196 of 8633 for the COVID-19 period, and 9334 of 10,535 for the post-endemic period. This corresponds to removal rates of 11.9%, 16.6%, and 11.4%, respectively, yielding a final cleaned corpus of 24,353 documents. Table 1 summarizes the corpus construction and cleaning results across the three analytical periods. Cleaning included the removal of duplicated, substantively irrelevant, or otherwise unusable textual records. Because document-level reason codes for exclusion were not retained, removal counts cannot be further disaggregated by exclusion category. The same preprocessing and keyword-selection criteria were applied across all three periods.

The cleaned datasets served as the basis for keyword extraction and selection, co-occurrence network construction, and cross-period comparison. Detailed procedures for text preprocessing and the selection of the 60 core keywords are described in Section 3.3, while the construction of the semantic networks and the centrality, CONCOR, and Jaccard similarity analyses are presented in Section 3.4. The overall research process is summarized in Figure 1.

### 3.3. Data Preprocessing and Core Keyword Selection

The collected online texts were cleaned and standardized to construct a consistent corpus suitable for examining the semantic structure of Korean temple food discourse. Duplicate or substantively uninformative documents, simple link collections, repetitive promotional phrases, advertisements, residual URL or HTML strings, and texts dominated by meaningless symbols or numbers were excluded from the analysis. Documents containing the search term “temple food” but lacking substantive relevance to Korean temple food, Buddhist food practices, food consumption, or related cultural and experiential contexts were also removed after their original context was reviewed. Expressions unrelated to the research topic, including terms associated with fortune-telling or shamanic initiation, were treated as noise. The same cleaning criteria were applied consistently across all three analytical periods to minimize the possibility that differences in preprocessing would affect the temporal comparison.

From the cleaned texts, major lexical items were extracted with a focus on nouns. Expressions representing the same lexical concept were standardized into common analytical units to reduce variation arising from grammatical or orthographic forms. For example, Korean expressions corresponding to “healthy,” “with history,” and “culture” with attached particles or endings were normalized to the noun forms “health,” “history,” and “culture,” respectively. In contrast, compound nouns and proper names carrying distinct food-related or cultural meanings were retained as single analytical units to preserve their semantic specificity. For example, Sandeulbada Temple Food was not decomposed into generic components but was retained as a proper-name node because it referred to an identifiable temple food restaurant, brand, and dining space.

The selection of core keywords was based on both frequency of occurrence and substantive relevance rather than frequency rank alone. High-frequency candidate terms were first identified from the word-frequency results for each period and were subsequently reviewed in their original contexts to determine whether they represented meaningful dimensions of Korean temple food discourse. These dimensions included Buddhist food culture, ingredients and culinary preparation, restaurant and food-service consumption, plant-based and vegetarian eating, health and wellness, cultural and experiential consumption, and related media or place-based contexts. This procedure was intended to retain terms that contributed substantively to the interpretation of the discourse while excluding words with limited discriminatory value for relational analysis.

Generic terms that largely duplicated the search concept or provided little analytical differentiation—such as “temple food,” “temple,” “food,” “Korea,” and “our country”—were therefore excluded from the final network nodes. In contrast, contextually meaningful terms capable of distinguishing food-related and period-specific meanings were retained. Examples included Dining Hotspot, Health, Vegetarianism, Veganism, Templestay, Balwoo Gongyang, Ven. Jeong Kwan, Ven. Seonjae, Healing, Meditation, Culinary Class Wars, and Culinary Master. Proper nouns were retained only when they represented identifiable actors, places, establishments, brands, or media-related entities that contributed substantively to the semantic structure of the discourse rather than functioning as incidental mentions.

A final set of 60 core keywords was selected separately for each of the three analytical periods. To enhance content validity, the appropriateness and substantive relevance of the selected keywords were cross-checked by two domain scholars specializing in foodservice and tourism. Differences in judgment were discussed, and the final keyword sets were determined through consensus. The 60-keyword cutoff was used to balance thematic coverage and network interpretability and to maintain a consistent network size across periods. Because corpus sizes differed, raw keyword frequencies were also normalized per 1000 retained documents to improve cross-period comparability.

The selected keywords were subsequently used for frequency analysis, semantic network construction, centrality analysis, network visualization, CONCOR clustering, and Jaccard similarity analysis. The complete 60-keyword sets and their period-specific frequencies are provided in Appendix Table A2. English labels used in the tables and figures represent standardized English glosses of the Korean keywords used in the original text analysis. Representative Korean forms and normalization examples are provided in Appendix Table A1. Sensitivity of the Jaccard results to alternative keyword-set sizes is examined separately in the robustness analysis.

### 3.4. Semantic Network, Centrality, CONCOR, and Jaccard Similarity Analyses

To examine the semantic structure of online discourse surrounding Korean temple food and its temporal changes across the COVID-19 transition, this study sequentially applied semantic network analysis, centrality analysis, CONCOR analysis, and Jaccard similarity analysis. Semantic network analysis extends beyond the frequency of individual keywords by representing relationships among words that appear within the same textual context. This approach makes it possible to examine not only which concepts are prominent but also how they are relationally embedded within the broader discourse. Co-occurrence-based network approaches have been used to identify relationships among major themes and concepts in large-scale textual data and to trace changes in semantic structures across different periods [55,56].

For each analytical period, the 60 selected core keywords were treated as nodes in a one-mode keyword network. Co-occurrence was defined at the document level: when two keywords appeared together in the same retained document, their joint presence was counted as one co-occurrence event regardless of repeated appearances within that document. These events were aggregated across all retained documents to construct a symmetric valued 60 × 60 co-occurrence matrix, with diagonal elements set to zero. This document-level co-word representation captures relational associations among keywords beyond their individual frequencies [57]. Comparable co-word approaches have been widely used to examine thematic relationships and temporal changes in large-scale textual data [58,59,60]. Because the number of retained documents differed across the three periods, raw co-occurrence counts were normalized to the number of co-occurring documents per 1000 retained documents. This normalization enabled more comparable assessment of relational intensity across periods with unequal corpus sizes.

Three complementary network measures were then calculated. Normalized weighted strength (NWS) represents the overall intensity of a keyword’s co-occurrence relationships and was calculated by summing its normalized co-occurrence weights. Degree centrality (DC) was calculated from the binary network, in which a tie was coded as present when two keywords co-occurred in at least one retained document. DC was normalized by the 59 possible direct ties and therefore represents the breadth of a keyword’s direct connectivity. Closeness centrality (CC) was calculated from shortest-path distances in the same binary network using normalized Freeman closeness and represents a keyword’s topological accessibility to the rest of the network. Thus, NWS captures relational intensity, whereas DC and CC capture connectivity breadth and network accessibility, respectively. Network analyses were conducted and verified using UCINET 6.847. To contextualize node-level centrality and assess broader differences in network connectedness across periods, network-level structural statistics were also calculated for each complete 60-node binary network. These included network density, average degree, average shortest-path length, diameter, and the number of isolates. These measures were used to determine whether changes in keyword-level centrality coincided with broader changes in the overall connectivity of the semantic networks. All network-level statistics were calculated using the complete 60 × 60 networks. For visualization only, Figure 2, Figure 3 and Figure 4 display the stronger normalized co-occurrence ties to improve graphical readability, whereas weaker ties were omitted from the displayed networks. This visual filtering did not affect the calculation of NWS, DC, CC, or any network-level structural statistics, all of which were based on the complete networks. Network visualizations were generated using Python 3.13.5 with NetworkX 3.6.1 and Matplotlib 3.10.8.

CONCOR (Convergence of Iterated Correlations) analysis was subsequently conducted to identify structurally equivalent semantic subgroups within each period-specific keyword network. Unlike clustering based solely on direct co-occurrence or keyword frequency, CONCOR groups nodes according to similarities in their relational profiles with other nodes in the network. Keywords assigned to the same CONCOR cluster can therefore be interpreted as occupying comparable structural positions within the broader semantic network [61,62]. For consistent cross-period interpretation, the CONCOR solutions were examined at a common four-cluster level for all three periods. The four-cluster specification was adopted as a comparative analytical framework and was not assumed to represent a statistically optimal community partition. To assess the robustness of this specification, the period-specific network structures were additionally compared with modularity-based community-detection results. These robustness analyses were used to evaluate whether the four-cluster CONCOR interpretation was broadly consistent with alternative structural partitions, with detailed results reported in Appendix Table A6. Cluster labels were assigned after examining the constituent keywords, their relational profiles, and the dominant semantic characteristics of each cluster. To reduce dependence on a single researcher’s interpretation, the proposed cluster labels were cross-checked by two domain scholars specializing in foodservice and tourism. Differences in interpretation were discussed, and the final cluster labels were determined through consensus. Finally, Jaccard similarity was calculated to quantify continuity and change in the composition of the core keyword sets across the three analytical periods. For two keyword sets *A* and *B*, the Jaccard similarity coefficient was calculated as:

$$J(A,B)=\frac{\left|A\cap B\right|}{\left|A\cup B\right|}$$

where *A* ∩ *B* represents the keywords shared by the two periods and *A* ∪ *B* represents all unique keywords appearing across the two sets. The coefficient ranges from 0 to 1, with higher values indicating greater overlap in keyword composition [63]. Jaccard similarity is particularly useful for comparing sets derived from different samples or temporally separated datasets because it standardizes the number of shared elements relative to the total number of unique elements in the two sets [64]. Previous studies have applied Jaccard-based measures to web text and longitudinal analyses to evaluate similarities and changes across different corpora or analytical periods [65,66,67].

In the present study, Jaccard similarity was calculated for three pairwise comparisons using the 60 core keywords selected according to the same criteria for each period: pre-COVID-19 versus COVID-19, COVID-19 versus post-endemic, and pre-COVID-19 versus post-endemic. The analysis was used to determine the extent to which the core vocabulary surrounding Korean temple food persisted or changed across the COVID-19 transition and to identify keywords that entered or dropped out of the period-specific sets. Importantly, the Jaccard coefficient was interpreted strictly as a measure of keyword-set overlap. It was not used as a measure of edge-weight similarity, network topology, or structural similarity between the full co-occurrence matrices. No fixed threshold was imposed to classify two periods as categorically similar or dissimilar. Instead, the relative magnitudes of the three pairwise coefficients were compared to assess the degree of continuity and reconfiguration in Korean temple food discourse over time. Because Jaccard similarity can be sensitive to the size of the selected keyword sets, cutoff sensitivity was additionally assessed using the top-40, top-60, and top-100 keyword sets for each analytical period. The robustness assessment focused on whether the relative ordering of pairwise similarity across the three period comparisons remained consistent under alternative keyword cutoffs.

### 3.5. Robustness and Sensitivity Analysis Procedures

To assess the stability of the network results under document-level sampling variation, a nonparametric bootstrap procedure was conducted separately for each analytical period. For each period, 1000 bootstrap samples were generated by resampling the retained documents with replacement while preserving the original period-specific sample size. The 60 validated keywords were held fixed, and the document-level co-occurrence matrix was reconstructed for each bootstrap sample. Normalized weighted strength (NWS), degree centrality (DC), closeness centrality (CC), network density, and average shortest-path length were recalculated for each iteration. The stability of node-level rankings was additionally assessed using Spearman rank correlations between the observed and bootstrap-derived rankings. Percentile-based 95% bootstrap intervals were used to summarize variation in the resampled network measures. Because Jaccard similarity based on fixed keyword sets is deterministic, a conventional bootstrap that holds keyword-set membership constant would not generate a meaningful sampling distribution. Therefore, sensitivity of the Jaccard results was assessed by repeating the analysis using alternative top-40, top-60, and top-100 keyword sets. Alternative cutoff sets were drawn from the same cleaned and standardized candidate-keyword lists prior to application of the final 60-keyword cutoff. This analysis examined whether the relative ordering of pairwise similarities remained stable across alternative cutoff levels rather than assuming that the absolute Jaccard coefficients were invariant to keyword-set size.

The robustness of the common four-cluster CONCOR interpretation was also examined using modularity-based community detection. Weighted Louvain community detection was repeated across 100 random starts, and greedy modularity solutions ranging from two to eight communities were compared with the four-cluster CONCOR structure. These analyses were used as robustness checks rather than replacements for CONCOR because the two approaches capture different structural principles: CONCOR emphasizes structural equivalence, whereas modularity-based methods identify comparatively dense communities. Detailed robustness and sensitivity results are reported in Appendix Table A4, Table A5 and Table A6.

## 4. Results

### 4.1. Frequency Analysis of Core Temple Food Keywords

Table 2 presents the 20 most frequently occurring keywords related to Korean temple food in the pre-COVID-19, COVID-19, and post-endemic periods. The complete set of 60 keywords and their frequencies for each period is provided in Appendix Table A2. Across the three periods, recurring terms were associated with restaurant and dining consumption, Buddhist food culture, plant-based eating, and experiential contexts, although their relative positions differed over time. Because the number of documents varied across the three periods, the results are interpreted primarily in terms of within-period keyword rankings and changes in keyword composition rather than direct comparisons of absolute frequencies across periods.

In the pre-COVID-19 period, Dining Hotspot (497) was the most frequent keyword, followed by Seoul (241), Vegetarianism (233), Restaurant (219), Food Preparation (217), Travel (206), Specialty Restaurant (204), Busan (193), Ingredients (180), and Eatery (179). The remainder of the top 20 consisted of Buddhist Temple (171), Experience (165), Temple Food Restaurant (165), Templestay (160), Cooking (159), Balwoo Gongyang (159), Buddhist Monk (148), Recommendation (144), Gyeonggi Province (142), and Tradition (140). Overall, the pre-pandemic keyword composition was strongly associated with restaurant and dining information while simultaneously reflecting the culinary characteristics of temple food. In particular, Vegetarianism, Food Preparation, Ingredients, Cooking, and Balwoo Gongyang occupied prominent positions, indicating that plant-based eating, preparation practices, ingredients, and traditional Buddhist food practices constituted important components of the discourse.

During the COVID-19 period, Dining Hotspot (651) remained the most frequent keyword, followed by Buddhist Temple (277), Seoul (243), Restaurant (243), Templestay (225), Temple Food Restaurant (222), Food Preparation (219), Vegetarianism (219), Buddhist Monk (216), and Gyeongju (210). Other top-20 keywords included Specialty Restaurant (202), Veganism (200), Travel (196), Recommendation (190), Tradition (170), Recipe (163), Baru (158), Busan (153), Cooking (146), and Eatery (140). Restaurant consumption, food preparation, and vegetarian eating therefore remained prominent, while Buddhist Temple, Templestay, and Buddhist Monk also occupied relatively high positions. A notable change was the appearance of Veganism among the top-ranked keywords. Together with the continued presence of Vegetarianism, this pattern indicates that contemporary plant-based eating became more visible within temple food discourse during the pandemic. Recipe, Food Preparation, and Cooking also remained prominent, reflecting continued attention to culinary preparation and food-related practices. Beyond the top 20, the broader 60-keyword set included COVID-19, Nature, Health, and Healing, showing that health- and lifestyle-related meanings were also present in the pandemic-period discourse (Appendix Table A2).

The post-endemic period exhibited a marked change in keyword composition. Travel (1330) became the most frequent term, followed by Dining Hotspot (1078), Recommendation (912), Buddhist Temple (595), Templestay (572), Buddhism (482), Seoul (478), Experience (468), Tradition (433), and Busan (433). The remaining top-20 keywords were Review (378), Places to Visit (362), Travel Course (362), Ven. Seonjae (317), Restaurant (315), Gyeongju (315), Nature (270), Veganism (266), Visit (265), and World (264). Compared with the earlier periods, terms related to information seeking, experience, and place-based consumption became considerably more visible, including Travel, Recommendation, Experience, Review, Places to Visit, Travel Course, and Visit. These changes indicate that temple food was increasingly discussed within broader experiential contexts that included food discovery, temple visits, Templestay, and regional mobility. Importantly, however, this expansion occurred alongside the continued presence of food- and culture-related terms such as Dining Hotspot, Restaurant, Veganism, Tradition, and Buddhism, suggesting that the established culinary and plant-based identity of temple food remained embedded within the expanding discourse.

The prominence of Ven. Seonjae (317) in the post-endemic top 20 also indicates greater visibility of identifiable culinary practitioners. The complete 60-keyword set additionally included Ven. Jeong Kwan, Culinary Master, Culinary Class Wars, Jingwansa Temple, and Bongnyeongsa Temple, reflecting the growing presence of practitioners, specific temple settings, and media-related content within contemporary temple food discourse (Appendix Table A2). These terms suggest that the circulation of temple food meanings increasingly involved not only food attributes and consumption settings but also recognizable experts, cultural institutions, and mediated representations.

Viewed across the three periods, the frequency results show both continuity and reconfiguration. A relatively stable vocabulary associated with dining, Buddhist food culture, vegetarian and plant-based eating, traditional culinary practices, and experiential consumption persisted across the observation period. The emphasis within this broader vocabulary nevertheless changed. Before COVID-19, the discourse placed comparatively greater emphasis on food preparation, ingredients, vegetarianism, and traditional Buddhist eating practices. During the pandemic, these themes remained visible while Veganism became more prominent and health-, nature-, and healing-related terms appeared within the broader keyword set. In the post-endemic period, information-seeking and experiential terms such as Travel, Recommendation, Experience, Review, Places to Visit, Travel Course, and Visit moved to more prominent positions, while Veganism, Nature, Buddhist culture, and restaurant-related terms continued to remain visible.

Overall, the frequency patterns indicate a gradual broadening of the semantic context surrounding Korean temple food rather than the replacement of its established culinary identity. The discourse evolved from a stronger emphasis on food preparation, ingredients, vegetarianism, and traditional Buddhist food practices before COVID-19, toward greater visibility of vegan and health-related meanings during the pandemic, and subsequently toward a more differentiated configuration incorporating experiential, place-based, wellness-related, and media-mediated meanings. These findings support the interpretation of Korean temple food as an evolving plant-based food culture whose traditional culinary foundations persisted while additional consumption and experiential meanings accumulated over time. However, frequency alone does not reveal the structural importance of individual keywords or the relational patterns connecting them. The following section therefore examines normalized weighted strength, degree centrality, and closeness centrality, together with the corresponding semantic network structures, before considering period-specific CONCOR clusters.

### 4.2. Network Centrality and Structural Characteristics

Table 3 presents normalized weighted strength (NWS), degree centrality (DC), and closeness centrality (CC) for the 20 most frequent keywords in each analytical period. Keywords are arranged according to their frequency ranks in Table 2 to facilitate comparison across analyses, while the complete network measures for all 60 keywords are provided in Appendix Table A3. NWS captures relational intensity, whereas DC and CC represent direct connectivity and network accessibility, respectively.

During the pre-COVID-19 period, Dining Hotspot showed the highest normalized weighted strength (NWS = 60.335) and also exhibited high degree centrality (DC = 0.915) and closeness centrality (CC = 0.922), indicating both strong relational intensity and broad network connectivity. Specialty Restaurant (NWS = 37.198), Vegetarianism (32.341), and Seoul (29.656) also occupied prominent positions, showing that the network was primarily structured around dining, plant-based eating, and urban food-related contexts.

During the COVID-19 period, Dining Hotspot remained the most prominent keyword in terms of relational intensity (NWS = 99.778), followed by Restaurant (49.055) and Specialty Restaurant (47.110). Dining Hotspot and Restaurant both showed high DC (0.898) and CC (0.908), indicating that dining- and restaurant-related concepts remained strongly embedded in the network. At the same time, Buddhist Temple, Templestay, Vegetarianism, and Veganism also occupied accessible positions, suggesting a broader integration of Buddhist cultural settings and plant-based eating within pandemic-period discourse.

In the post-endemic period, relational intensity was more strongly associated with tourism- and experience-related concepts. Travel (NWS = 177.844), Dining Hotspot (174.309), and Recommendation (169.809) showed the highest NWS values. Experience, Tradition, and Nature each recorded DC and CC values of 1.000, indicating direct connections with all 59 other keywords and complete topological accessibility within the binary network. Travel, Recommendation, Review, and Visit also occupied highly accessible positions, indicating that mobility, information seeking, and experiential consumption were strongly embedded in post-endemic temple food discourse.

At the network level, density increased from 0.586 in the pre-COVID-19 period to 0.649 during COVID-19 and 0.818 in the post-endemic period. Average degree likewise increased from 34.60 to 38.30 and 48.27, while average shortest-path length decreased from 1.416 to 1.351 and 1.182, respectively. Network diameter decreased from 3 in the pre-COVID-19 period to 2 during COVID-19 and remained at 2 in the post-endemic period, while no isolates were observed in any period. Taken together, these results indicate that the semantic network became progressively denser and more interconnected across the three periods. Because network density differed across periods, node-level network measures were interpreted primarily in terms of their structural roles within each period rather than as simple measures of temporal increase or decrease. The corresponding network visualizations are presented in Figure 2, Figure 3 and Figure 4.

### 4.3. CONCOR Analysis of Korean Temple Food Semantic Clusters

CONCOR analysis was conducted to identify semantic substructures within the broader networks of Korean temple food discourse. Rather than grouping keywords solely on the basis of direct co-occurrence, CONCOR classifies structurally equivalent keywords according to similarities in the relational patterns they form with other nodes. Four clusters were identified for each of the pre-COVID-19, COVID-19, and post-endemic networks. Cluster labels were assigned after examining the constituent keywords, their relational patterns, and their substantive relevance to Korean temple food discourse. To reduce dependence on a single researcher’s interpretation, the proposed labels were cross-checked by the same two domain scholars specializing in foodservice and tourism, and the final labels were determined through discussion and consensus. The results are presented in Table 4 and Figure 5, Figure 6 and Figure 7.

In the pre-COVID-19 period, the network was organized into four clusters: (1) Traditional Temple Food and Buddhist Dining Culture, (2) Vegetarian and Vegan Dining in Regional Food-Service Contexts, (3) Traditional Ingredients, Cooking, and Plant-Based Foods, and (4) Templestay and Buddhist Cultural Experience.

Cluster 1, “Traditional Temple Food and Buddhist Dining Culture,” included Balwoo Gongyang, Buddhist Offering, Korean Cuisine, Temple Cuisine, Hanjeongsik, Tradition, Buddhist Monk, Buddha, and Ven. Jeong Kwan. These keywords formed a semantic domain centered on traditional temple cuisine, Buddhist dietary practices, offering culture, and Korean culinary traditions. The grouping of Balwoo Gongyang, Buddhist Offering, Buddhist Monk, and Buddha indicates that temple food discourse before COVID-19 was embedded in Buddhist food practices rather than being limited to restaurant consumption. The presence of Korean Cuisine, Temple Cuisine, Hanjeongsik, and Tradition within the same cluster further positioned temple food within the broader context of traditional Korean food culture.

Cluster 2, “Vegetarian and Vegan Dining in Regional Food-Service Contexts,” comprised Vegetarianism, Veganism, Dining Hotspot, Restaurant, Specialty Restaurant, Temple Food Restaurant, Temple Food Specialty Restaurant, Cafe, Seoul, Busan, Gangwon Province, and Recommendation. This cluster reflects the intersection of plant-based eating with restaurant consumption and geographically situated food information. The grouping of Dining Hotspot, Restaurant, and Specialty Restaurant with regional terms such as Seoul, Busan, and Gangwon Province indicates that temple food was frequently discussed in relation to restaurant search and localized dining experiences. The presence of Vegetarianism and Veganism in the same relational structure further shows that plant-based meanings were already embedded in pre-pandemic temple food discourse alongside its traditional Buddhist identity.

Cluster 3, “Traditional Ingredients, Cooking, and Plant-Based Foods,” consisted primarily of Ingredients, Food Preparation, Cooking, Soy Sauce, Doenjang, Tofu, Kimchi, Jangajji, Namul, Mushrooms, Vegetables, Seasonal Ingredients, Five Pungent Vegetables, and Lotus Leaf Rice. This cluster represents a distinct culinary domain centered on ingredients, food preparation, and traditional plant-based dishes. The grouping of Soy Sauce, Doenjang, Tofu, Kimchi, Jangajji, and Namul with Seasonal Ingredients, Vegetables, and Mushrooms reflects the plant-based and seasonal composition of temple cuisine. The inclusion of Five Pungent Vegetables is particularly distinctive because it represents a culinary principle that differentiates Korean Buddhist temple cuisine from vegetarian eating more generally.

Cluster 4, “Templestay and Buddhist Cultural Experience,” included Buddhist Temple, Templestay, Experience, Travel, Culture, Gyeongju, Jeju, Jingwansa Temple, Bongnyeongsa Temple, and Ven. Seonjae. This cluster shows that temple food was already connected with temple settings, Templestay, travel, and cultural experience before COVID-19. The relational grouping of Buddhist Temple, Templestay, Experience, and Travel places temple food within a broader experiential and place-based setting associated with visiting temples and engaging with Buddhist culture. Nevertheless, the overall pre-pandemic cluster structure remained strongly organized around food, ingredients, preparation, dining, and Buddhist food practices, with experiential and travel-related meanings forming an additional contextual domain.

During the COVID-19 period, the network again formed four clusters: (1) Vegetarian and Vegan Temple Food Consumption, (2) Natural Ingredients, Cooking, and Health-Oriented Plant-Based Food, (3) Regional Travel, Healing, and Temple Food Discovery, and (4) Buddhist Culture, Templestay, and Traditional Food Practices.

Cluster 1, “Vegetarian and Vegan Temple Food Consumption,” included Vegetarianism, Veganism, Dining Hotspot, Restaurant, Specialty Restaurant, Temple Food Restaurant, Cafe, Seoul, Menu, Meal, Hanjeongsik, and Korean Cuisine. This cluster retained considerable continuity with the pre-COVID-19 plant-based dining cluster, while Veganism became more clearly embedded in the food-consumption structure during the pandemic. Temple food therefore continued to be associated with traditional Buddhist and Korean cuisine while simultaneously occupying a semantic domain linked to contemporary vegetarian and vegan eating.

Cluster 2, “Natural Ingredients, Cooking, and Health-Oriented Plant-Based Food,” contained Cooking, Recipe, Food Preparation, Ingredients, Soy Sauce, Doenjang, Tofu, Kimchi, Mushrooms, Seasonal Ingredients, Nature, Health, Temple Cuisine, and Lotus Leaf Rice. The ingredients-and-cooking structure identified before COVID-19 remained visible, but the inclusion of Nature and Health alongside food-preparation and ingredient terms represented a notable change in the cluster composition. This configuration indicates that the natural ingredients and culinary practices associated with temple food were increasingly discussed together with health-related meanings during the pandemic. The cluster therefore extended beyond culinary preparation alone to incorporate naturalness, health orientation, and plant-based eating.

Cluster 3, “Regional Travel, Healing, and Temple Food Discovery,” comprised Travel, Recommendation, Review, Places to Visit, Healing, Busan, Gyeongju, Jeju, Gangwon Province, Jeonnam Province, and Buddhist Temple. The grouping indicates that information surrounding temple food was also embedded in regional travel, recommendations, reviews, place discovery, and healing-related contexts. Recommendation, Review, and Places to Visit appeared together with Travel, Healing, regional place names, and Buddhist Temple, forming a distinct experiential and place-based semantic domain. Compared with the more food- and restaurant-centered structure of the pre-COVID-19 period, these meanings became more clearly differentiated during the pandemic.

Cluster 4, “Buddhist Culture, Templestay, and Traditional Food Practices,” included Templestay, Buddhist Monk, Buddhism, Culture, Tradition, Experience, Ven. Jeong Kwan, Bongnyeongsa Temple, Baekyangsa Temple, Balwoo Gongyang, and Buddhist Offering. This cluster demonstrates the continued relational presence of Buddhist culture, temple settings, Templestay, and traditional offering practices during the pandemic. The grouping of Templestay, Experience, Tradition, and Culture with Balwoo Gongyang and Buddhist Offering indicates that traditional Buddhist food practices remained embedded within broader cultural and experiential contexts.

In the post-endemic period, the semantic structure became more differentiated, forming four clusters: (1) Plant-Based Temple Cuisine, Health, and Traditional Food Practices, (2) Regional Travel, Reviews, and Healing Experiences, (3) Templestay, Buddhist Culture, and Meditation Experience, and (4) Culinary Experts, Media, and Temple Food Preparation.

Cluster 1, Plant-Based Temple Cuisine, Health, and Traditional Food Practices,” included Vegetarianism, Veganism, Korean Cuisine, Health, Seasonal Ingredients, Lotus Leaf Rice, Balwoo Gongyang, Sandeulbada Temple Food, and Five Pungent Vegetables. This cluster demonstrates that the identity of temple food as a plant-based food culture and traditional cuisine remained clearly represented after the endemic transition. The structural grouping of Vegetarianism, Veganism, and Health with traditional temple food-related terms indicates that contemporary plant-based and health-oriented meanings were incorporated into, rather than substituted for, its traditional Buddhist culinary identity.

Cluster 2, “Regional Travel, Reviews, and Healing Experiences,” comprised Travel, Recommendation, Review, Places to Visit, Travel Course, Travel Destination, Visit, Tourism, Local Area, Walking, Healing, Family, Busan, Gyeongju, and Jeju. Travel and place-based experience formed a particularly distinct semantic domain in the post-endemic network. The grouping of Travel, Recommendation, Review, Places to Visit, Travel Course, and Travel Destination demonstrates that temple food discourse became closely connected with travel planning, information search, and place discovery. The inclusion of Healing, Walking, and Family further shows that these discussions extended beyond food consumption itself into broader experiential and restorative contexts. Importantly, this cluster represents one contextual domain within the larger temple food network rather than indicating that tourism replaced the food-centered identity identified in Cluster 1.

Cluster 3, “Templestay, Buddhist Culture, and Meditation Experience,” contained Templestay, Experience, Meditation, Reservation, Buddhist Temple, Buddhism, Buddha, Buddhist Offering, Jingwansa Temple, Bongnyeongsa Temple, and Seoul. The Buddhist culture and Templestay structure identified during the pandemic remained evident, while Meditation and Reservation represented distinctive post-endemic elements. Their presence indicates that temple-related experiences were discussed not only in cultural terms but also through concrete participation and reservation practices alongside contemplative activities such as meditation. This pattern is consistent with the centrality results, in which Meditation and Reservation showed relatively high CC despite their lower frequency ranks. Together, these findings place temple food within an experiential setting in which Buddhist culture, participation, and wellness-related meanings coexist.

Cluster 4, “Culinary Experts, Media, and Temple Food Preparation,” included Ven. Seonjae, Ven. Jeong Kwan, Culinary Master, Culinary Class Wars, Buddhist Monk, Cooking, Food Preparation, Ingredients, Bibimbap, and Baekyangsa Temple. This cluster was distinctive because identifiable culinary practitioners, food-preparation terms, and media-related keywords formed an independent semantic domain in the post-endemic network. The grouping of Ven. Seonjae and Ven. Jeong Kwan with Culinary Master and Culinary Class Wars indicates that contemporary temple food discourse increasingly incorporated expert-led and media-mediated forms of food communication and popularization. Rather than indicating the displacement of traditional culinary culture, this configuration represents an additional pathway through which temple food and its preparation practices were circulated to broader audiences.

Taken together, the CONCOR results reveal continuity accompanied by gradual semantic differentiation. Before COVID-19, the network was organized primarily around traditional Buddhist food culture, plant-based dining, ingredients and cooking, and Templestay-related cultural experiences. During the pandemic, these established food and cultural domains remained evident, while Veganism, Health, Nature, and Healing became more explicitly integrated into the cluster structure. In particular, the simultaneous emergence of a vegetarian–vegan consumption cluster and a natural-ingredient and health-oriented plant-based food cluster demonstrates that contemporary plant-based and health-related meanings became more clearly differentiated within temple food discourse during this period.

As shown in Figure 7, the food-related core remained clearly visible through the cluster representing Plant-Based Temple Cuisine, Health, and Traditional Food Practices, while regional travel and healing-related experiences, Templestay and meditation, and expert-, media-, and food-preparation-related meanings formed separate semantic domains. Thus, the post-endemic discourse did not represent a simple shift away from food toward tourism or experience. Rather, a persistent plant-based and traditional culinary identity coexisted with increasingly differentiated experiential, wellness-related, place-based, and mediated meanings.

Overall, the CONCOR findings demonstrate that temporal change involved not merely fluctuations in individual keyword frequencies but also changes in the composition and organization of semantic substructures. Before COVID-19, traditional food, ingredients, cooking, plant-based dining, and Buddhist food culture constituted the principal domains. During the pandemic, vegetarianism, veganism, health, naturalness, and healing became more explicitly incorporated into the network. In the post-endemic period, the plant-based food identity remained distinct while experiential, wellness-related, place-based, and media-mediated meanings became increasingly differentiated. These findings support an interpretation of Korean temple food as an evolving Buddhist plant-based food culture whose traditional culinary foundations persisted while additional contemporary meanings accumulated and reorganized around them.

### 4.4. Jaccard Similarity Analysis of Core Korean Temple Food Keywords Across Periods

Jaccard similarity analysis was conducted using the 60-keyword sets from each analytical period to quantify continuity and change in the composition of Korean temple food discourse. The Jaccard coefficient represents the number of keywords shared by two sets relative to the total number of unique keywords contained in their union. Values closer to 1 indicate greater overlap in keyword composition, whereas values closer to 0 indicate greater compositional difference. As specified in Section 3.4, the coefficient was interpreted strictly as a measure of keyword-set overlap rather than as an indicator of structural similarity between the semantic networks. The results are presented in Table 5.

The comparison between the pre-COVID-19 and COVID-19 periods showed 51 shared keywords and 69 unique keywords across the two sets, yielding the highest Jaccard similarity among the three pairwise comparisons (J = 0.739). Thus, 51 of the 60 keywords in each period (85.0%) were shared, indicating substantial continuity in the core vocabulary surrounding Korean temple food despite the onset of the pandemic. Common keywords included Dining Hotspot, Vegetarianism, Restaurant, Buddhist Temple, Templestay, Cooking, Ingredients, Balwoo Gongyang, Buddhism, Buddhist Monk, Tradition, and Travel. These shared terms spanned restaurant consumption, plant-based eating, food preparation, Buddhist food culture, and experiential contexts.

At the same time, changes were evident within this relatively stable vocabulary. Keywords newly included in the COVID-19 top-60 set included COVID-19, Healing, Nature, Places to Visit, Baekyangsa Temple, Jeonnam Province, Dusu Gobang, Meal Table, and World. In particular, the inclusion of Healing and Nature indicates that wellness- and nature-related meanings became sufficiently prominent to enter the core keyword set during the pandemic. The relatively high Jaccard coefficient therefore reflects continuity without implying stasis: most of the pre-existing vocabulary remained, while additional health-, nature-, place-, and experience-related terms became incorporated into the selected keyword composition.

The comparison between the COVID-19 and post-endemic periods revealed 45 shared keywords and 75 unique keywords, resulting in a lower Jaccard similarity of J = 0.600. Although 45 of the 60 keywords in each set (75.0%) were shared, the compositional difference was greater than that observed between the pre-COVID-19 and COVID-19 periods. Travel, Dining Hotspot, Recommendation, Buddhist Temple, Templestay, Experience, Tradition, Veganism, Nature, Healing, Review, and Places to Visit remained common to both periods, demonstrating continued visibility of dining, Buddhist culture, plant-based eating, experiential consumption, and wellness-related meanings.

In the post-endemic period, Travel Course, Travel Destination, Tourism, Visit, Walking, Reservation, Meditation, Family, Local Area, History, Culinary Master, Culinary Class Wars, Bibimbap, Ven. Seonjae, and Jingwansa Temple entered the top-60 set. These additions indicate increasing differentiation in the contexts surrounding temple food. Travel Course, Travel Destination, Tourism, Visit, and Reservation reflect more explicit place-based and participation-oriented contexts; Meditation and Walking represent wellness- and activity-related meanings; and Culinary Master and Culinary Class Wars indicate the growing visibility of culinary expertise and media-mediated food discourse. The inclusion of Bibimbap also demonstrates that specific food-related terms remained part of the changing post-endemic vocabulary.

Conversely, several keywords included in the COVID-19 top-60 set were no longer included among the post-endemic top 60, including Doenjang, Soy Sauce, Tofu, Kimchi, Jangajji, Namul, Mushrooms, Vegetables, Recipe, Temple Cuisine, and Temple Food Restaurant. This pattern should not be interpreted as evidence that traditional ingredients or culinary practices disappeared from online discourse. Jaccard analysis captures only membership in the selected 60-keyword sets; therefore, a keyword dropping out of the top 60 indicates a change in its relative prominence within the selected vocabulary rather than its complete absence from the underlying corpus. Instead, experiential, place-based, wellness-related, and media-related terms became sufficiently prominent to alter the composition of the post-endemic core keyword set. This pattern is consistent with the frequency, centrality, and CONCOR results, which showed increasing differentiation of experiential and mediated meanings alongside the continuing presence of food-related themes.

The longest-term comparison, between the pre-COVID-19 and post-endemic periods, produced the lowest similarity. The two periods shared 41 keywords, while their union contained 79 unique keywords, resulting in a Jaccard similarity of J = 0.519. Shared terms included Dining Hotspot, Vegetarianism, Veganism, Buddhist Temple, Templestay, Cooking, Ingredients, Balwoo Gongyang, Buddhism, Buddhist Monk, Tradition, Experience, Travel, Recommendation, Culture, and Health. Their persistence demonstrates that a recognizable vocabulary associated with Buddhist food culture, plant-based eating, dining, culinary practice, and experiential meanings remained present across the full analytical period.

At the same time, the lower Jaccard coefficient indicates greater compositional reconfiguration over the longer interval. Keywords more characteristic of the pre-COVID-19 top-60 set included Garlic, Gochujang, Doenjang, Soy Sauce, Tofu, Kimchi, Jangajji, Namul, Mushrooms, Recipe, and Temple Cuisine, reflecting a comparatively strong representation of ingredients, cooking, and traditional food practices. By contrast, the post-endemic set included Travel Course, Travel Destination, Tourism, Visit, Walking, Reservation, Meditation, Family, Local Area, Culinary Master, and Culinary Class Wars. This contrast indicates that the selected vocabulary expanded beyond its strong culinary and ingredient-related orientation to incorporate more differentiated experiential, wellness-related, place-based, and media-mediated meanings.

A comparison across all three periods further illustrates the coexistence of continuity and change. Thirty-nine keywords were included in all three 60-keyword sets. These included Dining Hotspot, Vegetarianism, Veganism, Buddhist Temple, Templestay, Buddhist Monk, Buddhism, Cooking, Food Preparation, Ingredients, Balwoo Gongyang, Tradition, Experience, Travel, Recommendation, Health, and Culture. The persistence of these terms indicates that the discourse retained a stable lexical foundation associated with Buddhist food culture, plant-based eating, food preparation, dining, and culturally embedded experience throughout the COVID-19 transition.

Taken together, the pairwise Jaccard results indicate gradual compositional reconfiguration rather than an abrupt lexical break. Similarity was highest between the pre-COVID-19 and COVID-19 periods (J = 0.739), declined between the COVID-19 and post-endemic periods (J = 0.600), and was lowest between the pre-COVID-19 and post-endemic periods (J = 0.519). This pattern of pairwise similarity indicates greater compositional differentiation across the more temporally distant comparisons, while substantial overlap persisted across all three periods. The Jaccard results therefore complement the preceding frequency, centrality, and CONCOR analyses by showing that the traditional Buddhist and plant-based food vocabulary of Korean temple food remained visible while health-, wellness-, experiential-, place-based, and media-related terms became increasingly incorporated into the broader online discourse. The results support an interpretation of continuity accompanied by gradual recontextualization rather than the replacement of an established food identity.

### 4.5. Results of Robustness and Sensitivity Analyses

Because Jaccard similarity is deterministic for fixed keyword sets, conventional bootstrap confidence intervals were not estimated. Instead, robustness was assessed using alternative top-40, top-60, and top-100 keyword sets. Although the absolute Jaccard coefficients varied across cutoffs, their relative ordering remained unchanged. Similarity was consistently highest between the pre-COVID-19 and COVID-19 periods, followed by the COVID-19–post-endemic comparison, and lowest between the pre-COVID-19 and post-endemic periods. This consistent ordering indicates that the interpretation of temporal continuity and differentiation was robust to alternative keyword-set sizes (Appendix Table A4).

Document-level bootstrap analyses based on 1000 resamples per period further indicated high stability in the network results. Median Spearman correlations for NWS rankings were 0.953, 0.961, and 0.981 for the pre-COVID-19, COVID-19, and post-endemic periods, respectively, and the bootstrap intervals for increases in network density did not include zero. Modularity-based checks also showed that the four-cluster specification was strongly supported during COVID-19 and was near-optimal before COVID-19, whereas a three-community macrostructure was slightly more prominent in the post-endemic network. Because CONCOR identifies structural equivalence whereas modularity-based methods identify densely connected communities, these findings were treated as complementary robustness evidence rather than as directly equivalent clustering solutions (Appendix Table A5 and Table A6).

## 5. Discussion

This study examined how Korean-language online discourse surrounding Korean temple food was reconfigured across the pre-COVID-19, COVID-19, and post-endemic periods. By integrating frequency analysis, normalized weighted strength (NWS), degree and closeness centrality, CONCOR, and Jaccard similarity, the study identified a consistent pattern of continuity accompanied by semantic differentiation. Across the three periods, Buddhist culinary traditions, plant-based eating, food preparation, and dining remained visible, while health-, wellness-, experiential-, place-based-, and media-related meanings became more differentiated within the broader discourse.

The findings suggest that Korean temple food retained a food-centered identity throughout the analytical period. Before COVID-19, discourse was strongly associated with vegetarian eating, ingredients, culinary preparation, restaurant consumption, and traditional Buddhist food practices. During the COVID-19 period, these meanings remained visible while Veganism, Health, Nature, and Healing became more prominent. This pattern is consistent with previous research identifying increased attention to vegan discourse during the pandemic [51], although the present study situates such meanings within a culturally specific Buddhist food context rather than general vegan consumption.

In the post-endemic period, tourism-, experience-, and information-related terms occupied more prominent relational positions, while food, Buddhist culture, tradition, and plant-based meanings remained structurally embedded. The simultaneous accessibility of Experience, Tradition, Nature, Buddhist Temple, Templestay, and food-related terms suggests that experiential meanings were layered onto, rather than substituted for, the established culinary identity of temple food. The denser and more interconnected post-endemic network further indicates that temple food discourse was associated with a wider range of mutually connected cultural, food, wellness, and experiential contexts.

The CONCOR and Jaccard results reinforce this interpretation. Period-specific clusters differentiated plant-based food, traditional culinary practices, Buddhist culture, experiential contexts, regional travel, wellness, and media-related themes, while a recognizable food-cultural foundation persisted across periods. Similarity between the core keyword sets was highest for the pre-COVID-19 and COVID-19 periods and lowest for the pre-COVID-19 and post-endemic periods, indicating gradual compositional differentiation rather than an abrupt lexical break. This interpretation is consistent with previous studies showing that contemporary meanings can coexist with established Buddhist culinary traditions [5,6,17] and with social big-data research identifying temporal reorganization in food-related online discourse [45,46].

Taken together, the findings address RQ4 by showing that changes in Korean temple food discourse are best understood as layered semantic reconfiguration. Its Buddhist and plant-based culinary foundation remained recognizable, while additional meanings related to wellness, experience, place, culinary expertise, and digital media became more visible and interconnected. Importantly, these temporal patterns describe changes in online discourse and should not be interpreted as evidence that COVID-19 itself directly caused the observed changes.

### 5.1. Theoretical Implications

This study contributes to research on traditional food, plant-based food culture, and digitally mediated food discourse in three ways. First, it supports the conceptualization of Korean temple food as a culturally embedded Buddhist plant-based food culture rather than solely as religious cuisine, vegetarian food, or a tourism experience. Its contemporary meaning combines Buddhist culinary principles, plant-based preparation, natural and seasonal ingredients, and traditional food practices with health- and experience-related meanings. This food-centered conceptualization helps explain how culturally specific dietary traditions can retain their historical foundations while being interpreted through contemporary consumption values.

Second, the findings conceptualize temporal change as continuity with layered semantic reconfiguration rather than replacement. Traditional culinary and Buddhist meanings remained visible across all three periods, while vegan, wellness-related, experiential, place-based, and media-mediated meanings became more differentiated. The post-endemic presence of both traditional food practices and experiential or media-related domains therefore suggests that contemporary food identities can expand through the accumulation and reorganization of meanings without necessarily losing their cultural foundations. This extends previous interpretations of temple food as a tradition that is continuously recontextualized within contemporary society [5,6,17].

Third, the study demonstrates the value of combining lexical and relational approaches for longitudinal food-discourse research. Frequency indicates the prominence of individual terms, NWS captures relational intensity, DC and CC represent connectivity and accessibility, CONCOR identifies structurally differentiated semantic domains, and Jaccard similarity assesses continuity in keyword composition. Their combined use provides a more multidimensional account of changing food discourse than frequency analysis alone. At the same time, these measures describe socially produced online meanings rather than individual consumer attitudes or behaviors, which distinguishes the present approach from survey-based consumer research.

### 5.2. Practical Implications

The findings suggest that Korean temple food should be communicated primarily as a distinctive form of Buddhist plant-based food culture. Communication strategies can emphasize plant-based preparation, seasonal and natural ingredients, moderation, traditional culinary practices, and Buddhist food philosophy while clearly distinguishing temple food from generic vegetarian or vegan cuisine. Health-related messages should focus on perceived food values and cultural meanings rather than making unsupported nutritional or therapeutic claims.

Second, food-service and educational organizations can connect this culinary identity with participatory experiences. Restaurants, temple food education centers, and Buddhist or cultural institutions may develop cooking demonstrations, ingredient interpretation, seasonal menus, and hands-on culinary programs that explain not only individual dishes but also the preparation principles and cultural meanings underlying temple food. Digital information concerning menus, locations, reservations, programs, and related cultural activities can also make these experiences easier to discover and access.

Third, practitioners, media, and experiential settings can broaden public engagement without displacing the food-centered identity of temple cuisine. The visibility of culinary experts, media-related terms, Templestay, meditation, and place-based activities indicates opportunities for expert-led content, digital media, temple visits, and cultural programs. Such applications should, however, preserve the authenticity of ingredients, preparation principles, terminology, and Buddhist cultural meanings. Experiential and tourism-related activities are therefore best understood as contexts through which temple food is communicated and experienced rather than as reasons to reposition it primarily as a tourism product.

### 5.3. Limitations and Future Research Directions

This study has several limitations. First, the analysis was based on Korean-language online discourse retrieved using the focal search term “사찰음식” from selected Korean platforms and Google-indexed Facebook-related documents. The findings therefore should not be generalized to global temple food discourse or to Korean consumers as a statistically representative population. The use of a single search term may also have excluded relevant discussions expressed through alternative terminology. Moreover, because historical content was collected retrospectively, platform policies, search indexing, content deletion, and source availability may have affected the composition of the period-specific corpora. Future research could use multiple search terms, platform-specific sampling, or prospectively archived data.

Second, semantic network analysis identifies keyword co-occurrence and relational structure but cannot fully determine contextual meaning, evaluative orientation, or communicative intention. Associations involving Veganism, Health, Healing, Meditation, Travel, or Recommendation, for example, do not reveal whether these terms appeared in positive or negative evaluations or in specific consumption contexts. Future studies could combine network analysis with sentiment analysis, topic modeling, contextual embedding, or qualitative content analysis.

Third, the observed temporal differences should not be interpreted as direct causal effects of COVID-19. The post-endemic period coincided with changes in mobility, media exposure, vegan and wellness-related food interest, and broader consumption patterns. The three periods therefore represent distinct temporal contexts rather than experimentally isolated pandemic effects. More fine-grained temporal designs, such as monthly or quarterly analysis, interrupted time-series analysis, or change-point detection, could help identify when specific discourse changes occurred.

Fourth, this study examined online discourse rather than actual food choices, consumption behavior, or objectively measured food characteristics. The prominence of terms such as Veganism, Healing, Meditation, or Travel does not demonstrate corresponding changes in dietary adoption, restaurant visits, program participation, or purchasing behavior. Likewise, references to Health or Healing represent meanings expressed in online discourse rather than evidence of nutritional or therapeutic effects. Future research could integrate social big data with consumer surveys, experiments, interviews, behavioral records, sensory evaluation, and nutritional analysis to connect online discourse with actual food perceptions, choices, and product characteristics.

## 6. Conclusions

This study demonstrates that online discourse surrounding Korean temple food across the COVID-19 transition was characterized by continuity accompanied by gradual recontextualization rather than replacement. Its Buddhist and plant-based culinary foundation remained visible across all three periods, while vegan, health-oriented, wellness-related, experiential, place-based, and media-mediated meanings became increasingly differentiated, particularly in the post-endemic period. The Jaccard results further indicate substantial lexical continuity alongside greater compositional differentiation across more temporally distant comparisons.

By integrating frequency, centrality, CONCOR, and Jaccard analyses, this study provides longitudinal evidence that a culturally embedded traditional food can acquire contemporary meanings without losing its culinary and religious foundations. Korean temple food can therefore be understood as an evolving Buddhist plant-based food culture within contemporary food discourse. Future research should connect these online semantic patterns with consumer behavior, sensory evaluation, and nutritional or food-quality evidence.

## Figures and Tables

**Figure 1:**
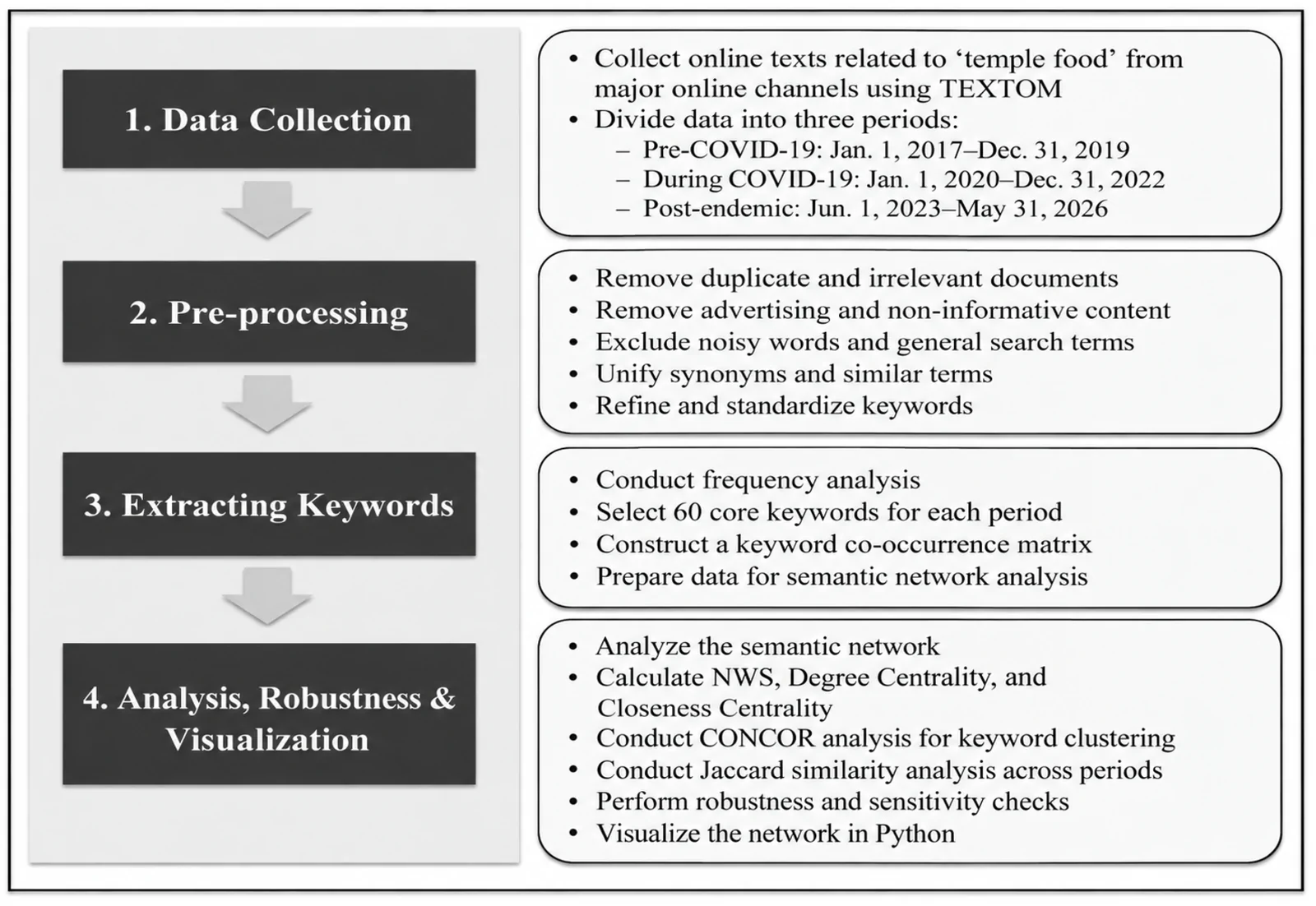
Research process for temple food semantic network analysis.

**Figure 2:**
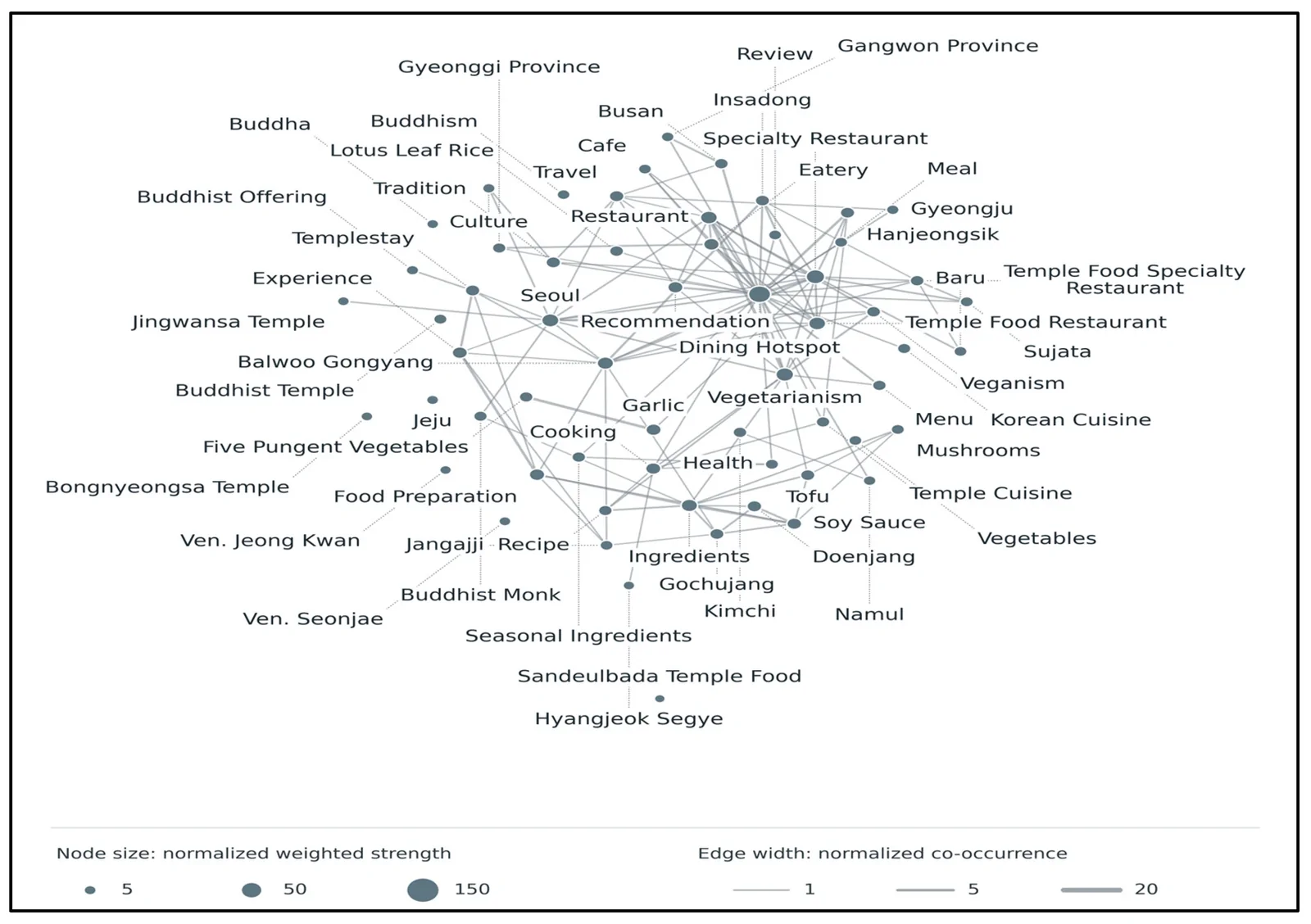
Semantic network of Korean temple food keywords in the pre-COVID-19 period. Node size represents normalized weighted strength and edge width normalized co-occurrence. Stronger ties are shown for readability.

**Figure 3:**
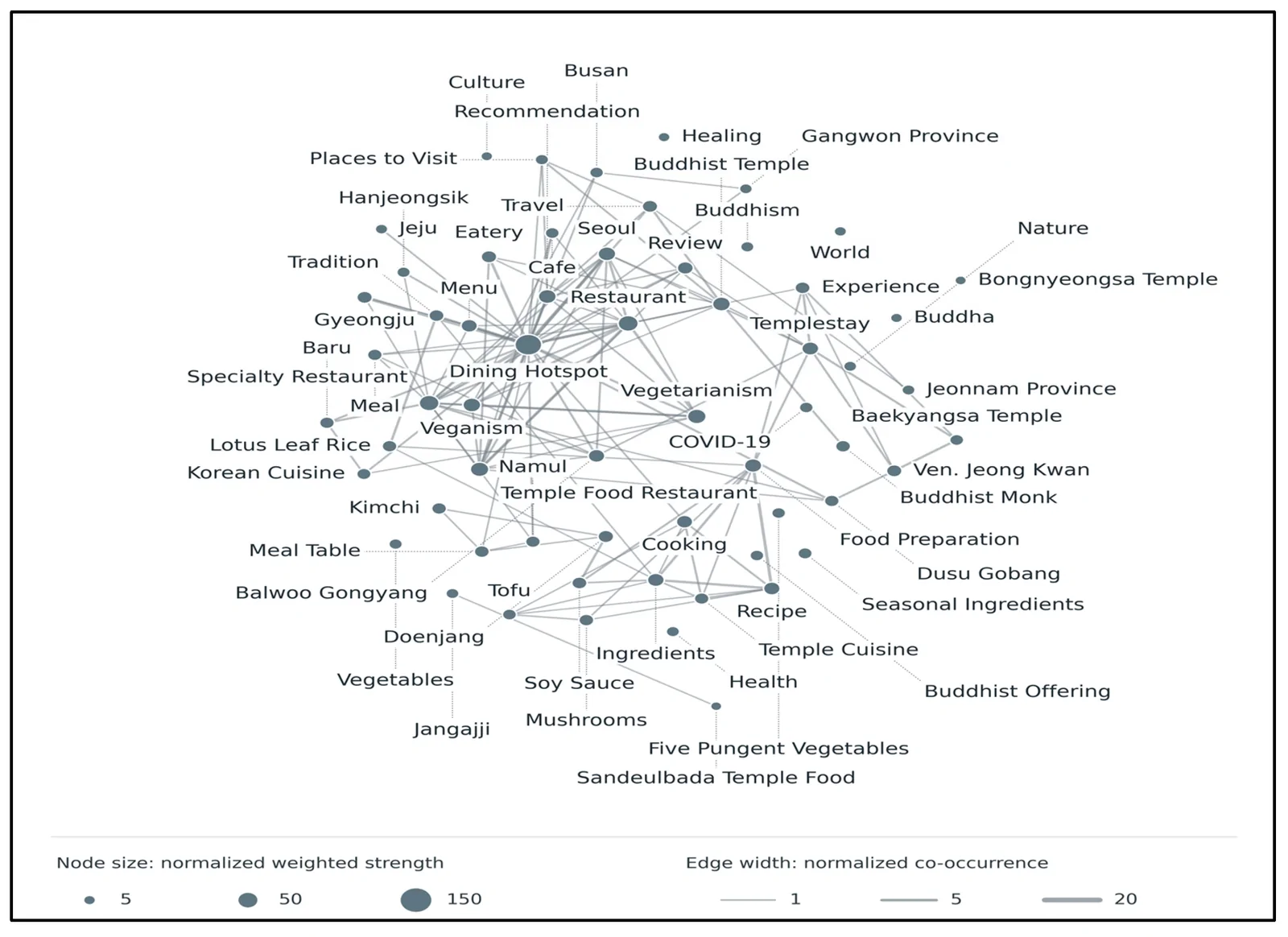
Semantic network of Korean temple food keywords during the COVID-19 period. Node size indicates normalized weighted strength and edge width indicates normalized co-occurrence. Only stronger ties are shown for readability; all network measures were calculated from the complete network.

**Figure 4:**
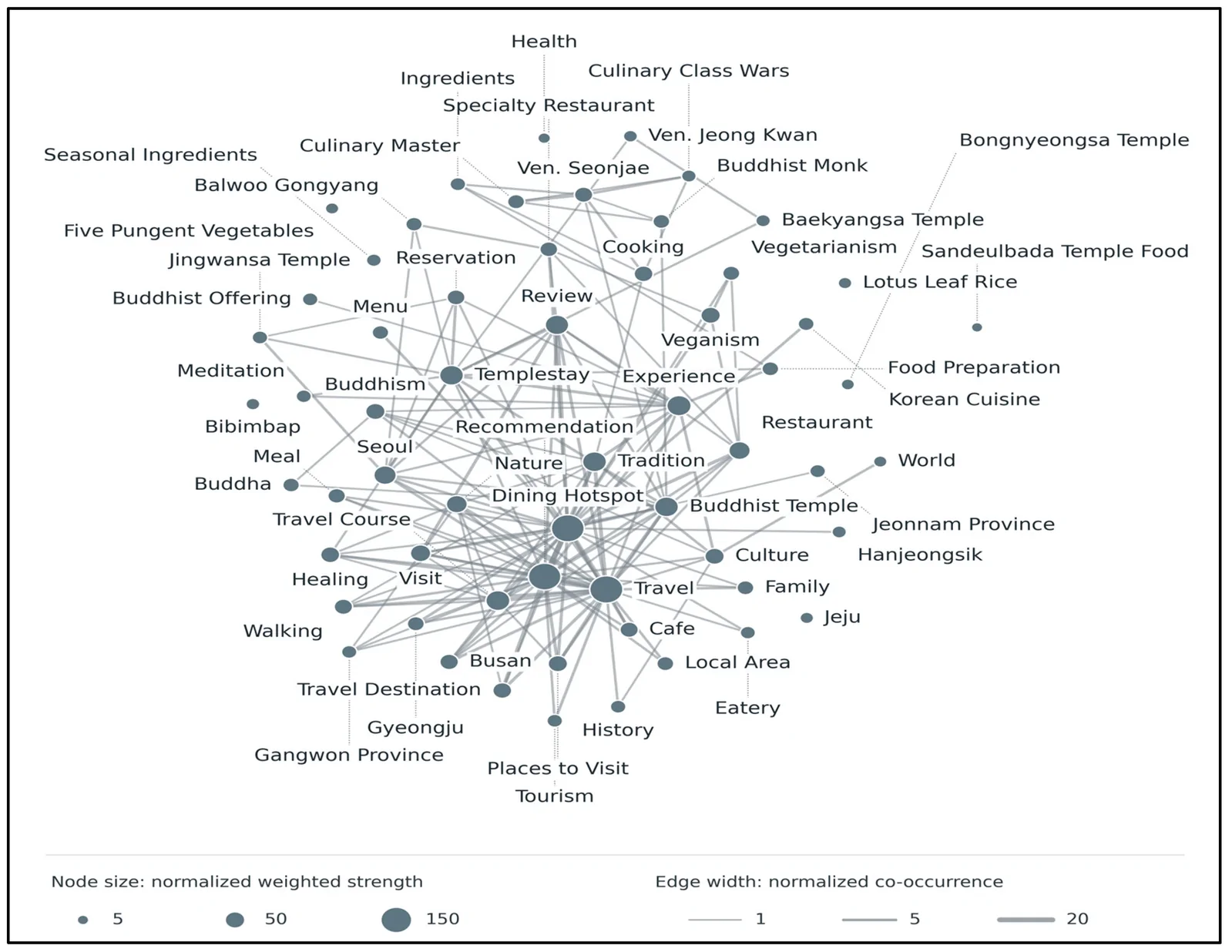
Semantic network of Korean temple food keywords in the post-endemic period. Node size represents normalized weighted strength and edge width normalized co-occurrence; stronger ties are shown for readability.

**Figure 5:**
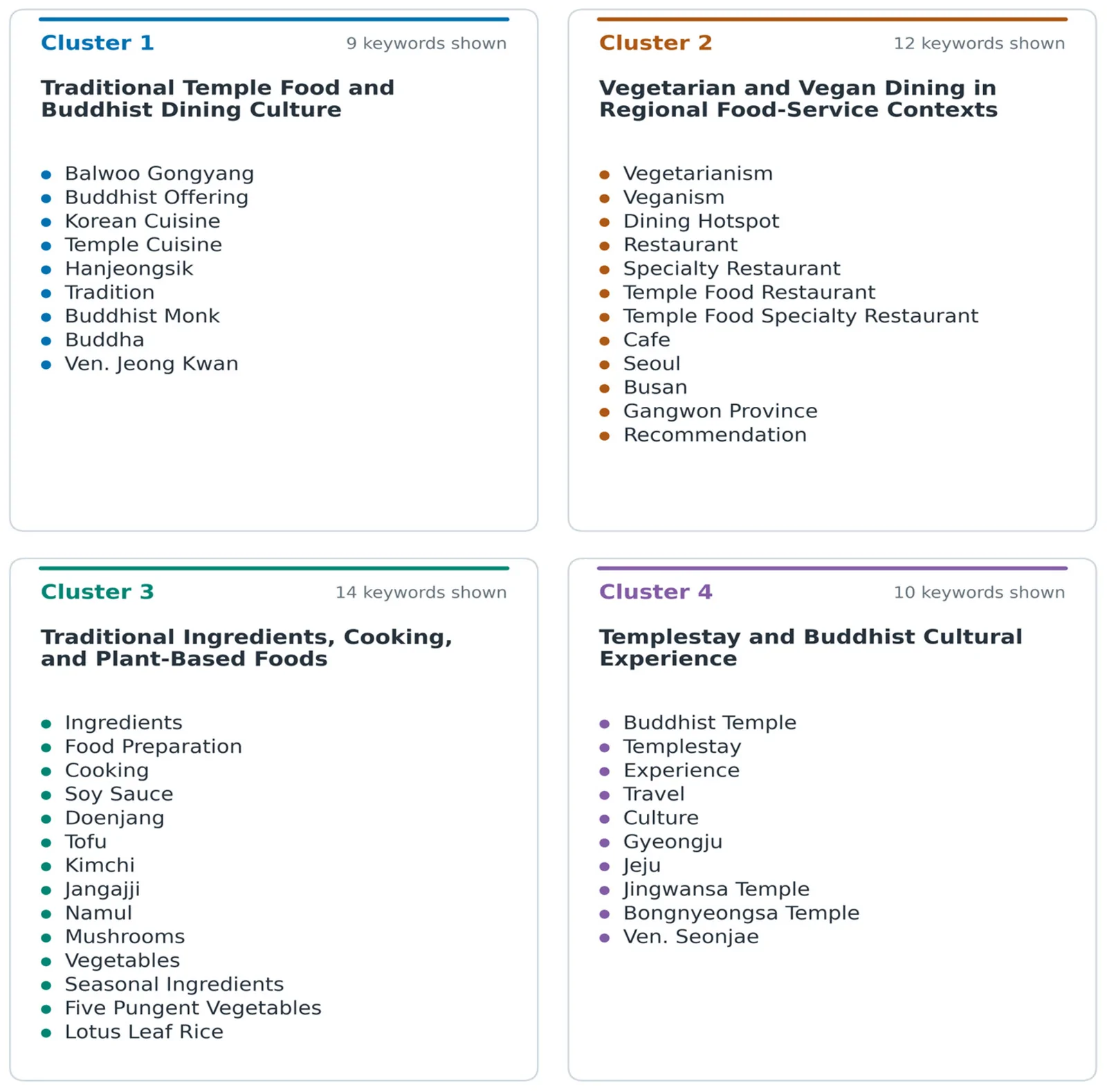
Representative keywords in CONCOR clusters: pre-COVID-19 period.

**Figure 6:**
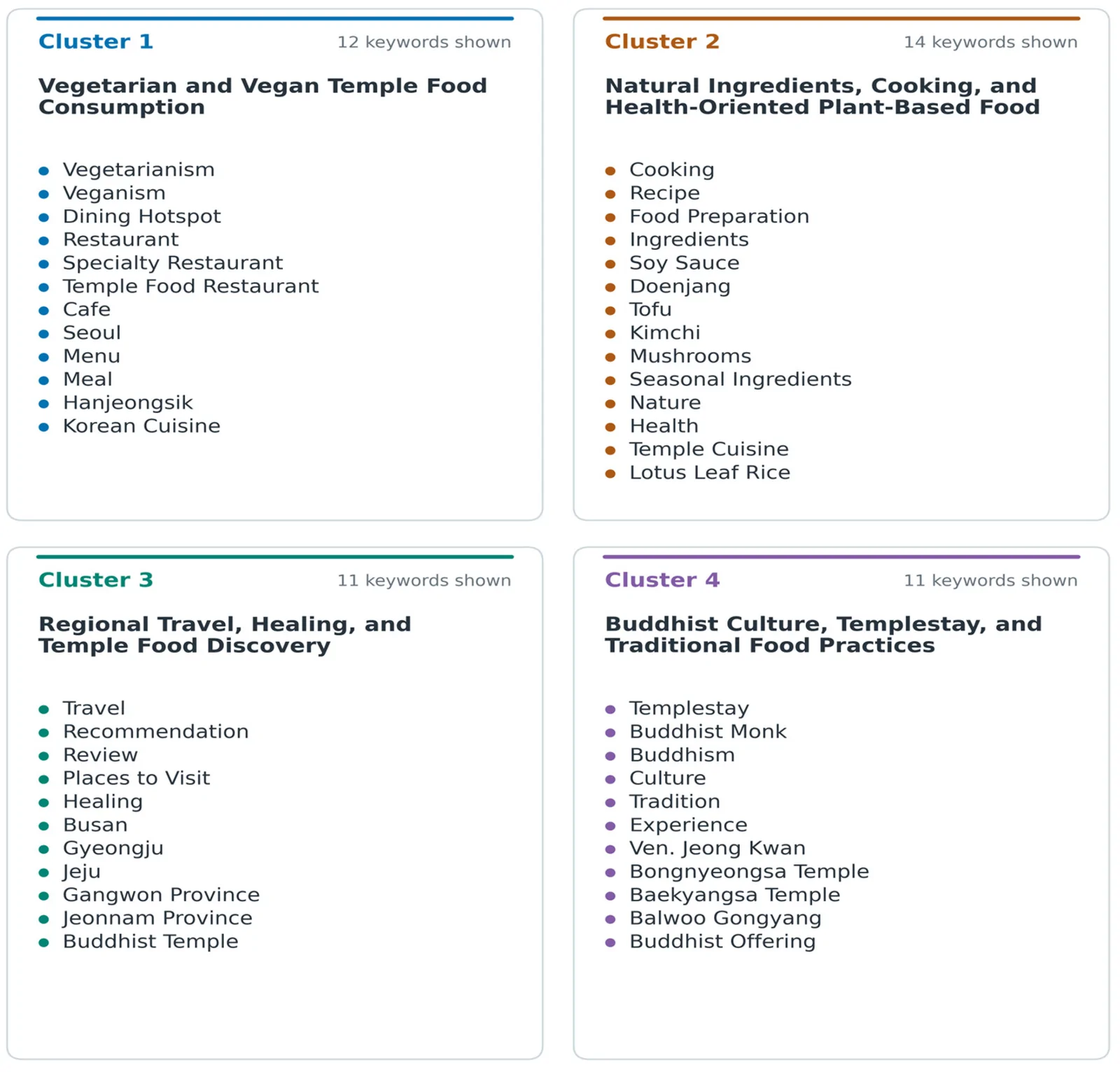
Representative keywords in CONCOR clusters: COVID-19 period.

**Figure 7:**
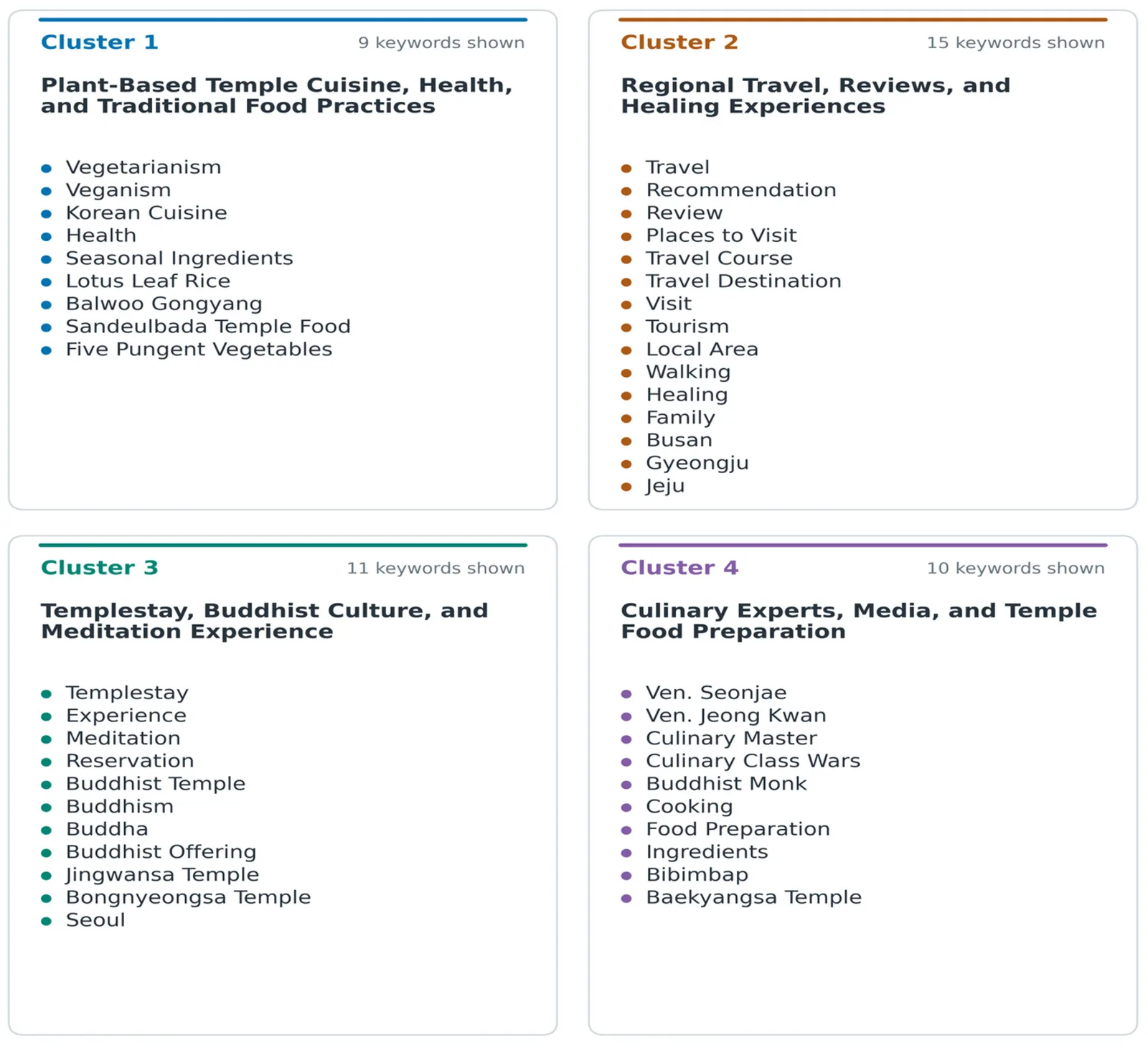
Representative keywords in CONCOR clusters: post-endemic period.

**Table 1:** Corpus construction and cleaning summary.

Corpus Construction Stage	Pre-COVID-19: 1 Jan. 2017–31 Dec. 2019	During COVID-19: 1 Jan. 2020–31 Dec. 2022	Post-Endemic: 1 Jun. 2023–31 May 2026
Raw documents collected	8880	8633	10,535
Documents removed during cleaning	1057	1437	1201
Final documents retained after cleaning	7823	7196	9334
Core keywords retained for network analysis	60	60	60

**Table 2:** Top 20 keywords and normalized frequencies by analytical period.

Rank	Period 1: Pre-COVID-19		Period 2: COVID-19		Period 3: Post-Endemic	
	Keyword	*n* (/1000)	Keyword	*n* (/1000)	Keyword	*n* (/1000)
1	Dining Hotspot	497 (63.5)	Dining Hotspot	651 (90.5)	Travel	1330 (142.5)
2	Seoul	241 (30.8)	Buddhist Temple	277 (38.5)	Dining Hotspot	1078 (115.5)
3	Vegetarianism	233 (29.8)	Seoul	243 (33.8)	Recommendation	912 (97.7)
4	Restaurant	219 (28.0)	Restaurant	243 (33.8)	Buddhist Temple	595 (63.7)
5	Food Preparation	217 (27.7)	Templestay	225 (31.3)	Templestay	572 (61.3)
6	Travel	206 (26.3)	Temple Food Restaurant	222 (30.9)	Buddhism	482 (51.6)
7	Specialty Restaurant	204 (26.1)	Food Preparation	219 (30.4)	Seoul	478 (51.2)
8	Busan	193 (24.7)	Vegetarianism	219 (30.4)	Experience	468 (50.1)
9	Ingredients	180 (23.0)	Buddhist Monk	216 (30.0)	Tradition	433 (46.4)
10	Eatery	179 (22.9)	Gyeongju	210 (29.2)	Busan	433 (46.4)
11	Buddhist Temple	171 (21.9)	Specialty Restaurant	202 (28.1)	Review	378 (40.5)
12	Experience	165 (21.1)	Veganism	200 (27.8)	Places to Visit	362 (38.8)
13	Temple Food Restaurant	165 (21.1)	Travel	196 (27.2)	Travel Course	362 (38.8)
14	Templestay	160 (20.5)	Recommendation	190 (26.4)	Ven. Seonjae	317 (34.0)
15	Cooking	159 (20.3)	Tradition	170 (23.6)	Restaurant	315 (33.7)
16	Balwoo Gongyang	159 (20.3)	Recipe	163 (22.7)	Gyeongju	315 (33.7)
17	Buddhist Monk	148 (18.9)	Baru	158 (22.0)	Nature	270 (28.9)
18	Recommendation	144 (18.4)	Busan	153 (21.3)	Veganism	266 (28.5)
19	Gyeonggi Province	142 (18.2)	Cooking	146 (20.3)	Visit	265 (28.4)
20	Tradition	140 (17.9)	Eatery	140 (19.5)	World	264 (28.3)

Note: Values are presented as raw document frequencies followed by normalized frequencies per 1000 retained documents in parentheses. Normalization accounts for differences in corpus size across periods. Keywords are ranked by raw frequency within each period; normalization does not alter within-period rankings.

**Table 3:** Normalized weighted strength, degree centrality, and closeness centrality of the top 20 Korean temple food keywords by analytical period. Panel (**A**). Pre-COVID-19 period. Panel (**B**). COVID-19 period. Panel (**C**). Post-endemic period.

(**A**)				
**Frequency Rank**	**Keyword**	**NWS**	**DC**	**CC**
1	Dining Hotspot	60.335	0.915	0.922
2	Seoul	29.656	0.881	0.894
3	Vegetarianism	32.341	0.864	0.881
4	Restaurant	27.867	0.729	0.787
5	Food Preparation	20.58	0.695	0.766
6	Travel	16.873	0.627	0.728
7	Specialty Restaurant	37.198	0.797	0.831
8	Busan	13.422	0.559	0.694
9	Ingredients	25.182	0.661	0.747
10	Eatery	20.964	0.746	0.797
11	Buddhist Temple	10.738	0.576	0.702
12	Experience	19.43	0.661	0.747
13	Temple Food Restaurant	28.378	0.814	0.843
14	Templestay	16.49	0.627	0.728
15	Cooking	19.302	0.797	0.831
16	Balwoo Gongyang	25.054	0.678	0.756
17	Buddhist Monk	11.888	0.644	0.738
18	Recommendation	19.302	0.763	0.808
19	Gyeonggi Province	11.888	0.542	0.686
20	Tradition	16.234	0.695	0.766
(**B**)				
**Frequency Rank**	**Keyword**	**NWS**	**DC**	**CC**
1	Dining Hotspot	99.778	0.898	0.908
2	Buddhist Temple	34.742	0.864	0.881
3	Seoul	33.769	0.797	0.831
4	Restaurant	49.055	0.898	0.908
5	Templestay	29.183	0.763	0.808
6	Temple Food Restaurant	38.494	0.763	0.808
7	Food Preparation	31.684	0.746	0.797
8	Vegetarianism	39.605	0.864	0.881
9	Buddhist Monk	19.038	0.729	0.787
10	Gyeongju	21.54	0.542	0.686
11	Specialty Restaurant	47.11	0.831	0.855
12	Veganism	34.603	0.763	0.808
13	Travel	21.818	0.644	0.738
14	Recommendation	35.019	0.864	0.881
15	Tradition	20.15	0.797	0.831
16	Recipe	27.515	0.627	0.728
17	Baru	18.066	0.525	0.678
18	Busan	14.036	0.508	0.67
19	Cooking	26.265	0.763	0.808
20	Eatery	22.374	0.678	0.756
(**C**)				
**Frequency Rank**	**Keyword**	**NWS**	**DC**	**CC**
1	Travel	177.844	0.966	0.967
2	Dining Hotspot	174.309	0.983	0.983
3	Recommendation	169.809	0.966	0.967
4	Buddhist Temple	83.351	0.949	0.952
5	Templestay	83.458	0.949	0.952
6	Buddhism	49.496	0.949	0.952
7	Seoul	71.888	0.932	0.937
8	Experience	87.958	1	1
9	Tradition	84.53	1	1
10	Busan	44.033	0.746	0.797
11	Review	80.673	0.966	0.967
12	Places to Visit	49.496	0.797	0.831
13	Travel Course	85.172	0.949	0.952
14	Ven. Seonjae	42.104	0.797	0.831
15	Restaurant	64.174	0.949	0.952
16	Gyeongju	34.176	0.797	0.831
17	Nature	60.531	1	1
18	Veganism	49.925	0.949	0.952
19	Visit	56.139	0.983	0.983
20	World	14.249	0.712	0.776

Note: Keywords are ordered according to their frequency ranks. NWS denotes normalized weighted strength per 1000 retained documents. DC denotes normalized degree centrality based on the binary adjacency matrix, and CC denotes normalized closeness centrality based on shortest-path distances in the same binary network.

**Table 4:** CONCOR Analysis of Korean Temple Food Semantic Clusters by Analytical Period.

Period	Cluster	Cluster Title	Representative Keywords
Period 1: Pre-COVID-19 Period	Cluster 1	Traditional Temple Food and Buddhist Dining Culture	Balwoo Gongyang, Buddhist Offering, Korean Cuisine, Temple Cuisine, Hanjeongsik, Tradition, Buddhist Monk, Buddha, Ven. Jeong Kwan
	Cluster 2	Vegetarian and Vegan Dining in Regional Food-Service Contexts	Vegetarianism, Veganism, Dining Hotspot, Restaurant, Specialty Restaurant, Temple Food Restaurant, Temple Food Specialty Restaurant, Cafe, Seoul, Busan, Gangwon Province, Recommendation
	Cluster 3	Traditional Ingredients, Cooking, and Plant-Based Foods	Ingredients, Food Preparation, Cooking, Soy Sauce, Doenjang, Tofu, Kimchi, Jangajji, Namul, Mushrooms, Vegetables, Seasonal Ingredients, Five Pungent Vegetables, Lotus Leaf Rice
	Cluster 4	Templestay and Buddhist Cultural Experience	Buddhist Temple, Templestay, Experience, Travel, Culture, Gyeongju, Jeju, Jingwansa Temple, Bongnyeongsa Temple, Ven. Seonjae
Period 2: COVID-19 Period	Cluster 1	Vegetarian and Vegan Temple Food Consumption	Vegetarianism, Veganism, Dining Hotspot, Restaurant, Specialty Restaurant, Temple Food Restaurant, Cafe, Seoul, Menu, Meal, Hanjeongsik, Korean Cuisine
	Cluster 2	Natural Ingredients, Cooking, and Health-Oriented Plant-Based Food	Cooking, Recipe, Food Preparation, Ingredients, Soy Sauce, Doenjang, Tofu, Kimchi, Mushrooms, Seasonal Ingredients, Nature, Health, Temple Cuisine, Lotus Leaf Rice
	Cluster 3	Regional Travel, Healing, and Temple Food Discovery	Travel, Recommendation, Review, Places to Visit, Healing, Busan, Gyeongju, Jeju, Gangwon Province, Jeonnam Province, Buddhist Temple
	Cluster 4	Buddhist Culture, Templestay, and Traditional Food Practices	Templestay, Buddhist Monk, Buddhism, Culture, Tradition, Experience, Ven. Jeong Kwan, Bongnyeongsa Temple, Baekyangsa Temple, Balwoo Gongyang, Buddhist Offering
Period 3: Post-Endemic Period	Cluster 1	Plant-Based Temple Cuisine, Health, and Traditional Food Practices	Vegetarianism, Veganism, Korean Cuisine, Health, Seasonal Ingredients, Lotus Leaf Rice, Balwoo Gongyang, Sandeulbada Temple Food, Five Pungent Vegetables
	Cluster 2	Regional Travel, Reviews, and Healing Experiences	Travel, Recommendation, Review, Places to Visit, Travel Course, Travel Destination, Visit, Tourism, Local Area, Walking, Healing, Family, Busan, Gyeongju, Jeju
	Cluster 3	Templestay, Buddhist Culture, and Meditation Experience	Templestay, Experience, Meditation, Reservation, Buddhist Temple, Buddhism, Buddha, Buddhist Offering, Jingwansa Temple, Bongnyeongsa Temple, Seoul
	Cluster 4	Culinary Experts, Media, and Temple Food Preparation	Ven. Seonjae, Ven. Jeong Kwan, Culinary Master, Culinary Class Wars, Buddhist Monk, Cooking, Food Preparation, Ingredients, Bibimbap, Baekyangsa Temple

Note: Cluster titles were assigned based on the dominant semantic characteristics of the keywords grouped through CONCOR analysis. Representative keywords illustrate the primary thematic composition of each cluster.

**Table 5:** Jaccard Similarity and Core Keyword Changes Across Analytical Periods.

Period Comparison	Shared Keywords	Newly Included	Dropped	Union	Jaccard Similarity
Pre-COVID-19 vs. COVID-19	51	9	9	69	0.739
COVID-19 vs. Post-Endemic	45	15	15	75	0.600
Pre-COVID-19 vs. Post-Endemic	41	19	19	79	0.519

Note. Shared Keywords refers to keywords included in the top-60 sets of both periods. Newly Included refers to keywords entering the subsequent period’s top-60 set, whereas Dropped refers to keywords included in the earlier period’s top-60 set but not in the subsequent period’s set. Jaccard similarity represents keyword-set overlap and does not indicate similarity in network topology or edge structure.

**Figure A1:**
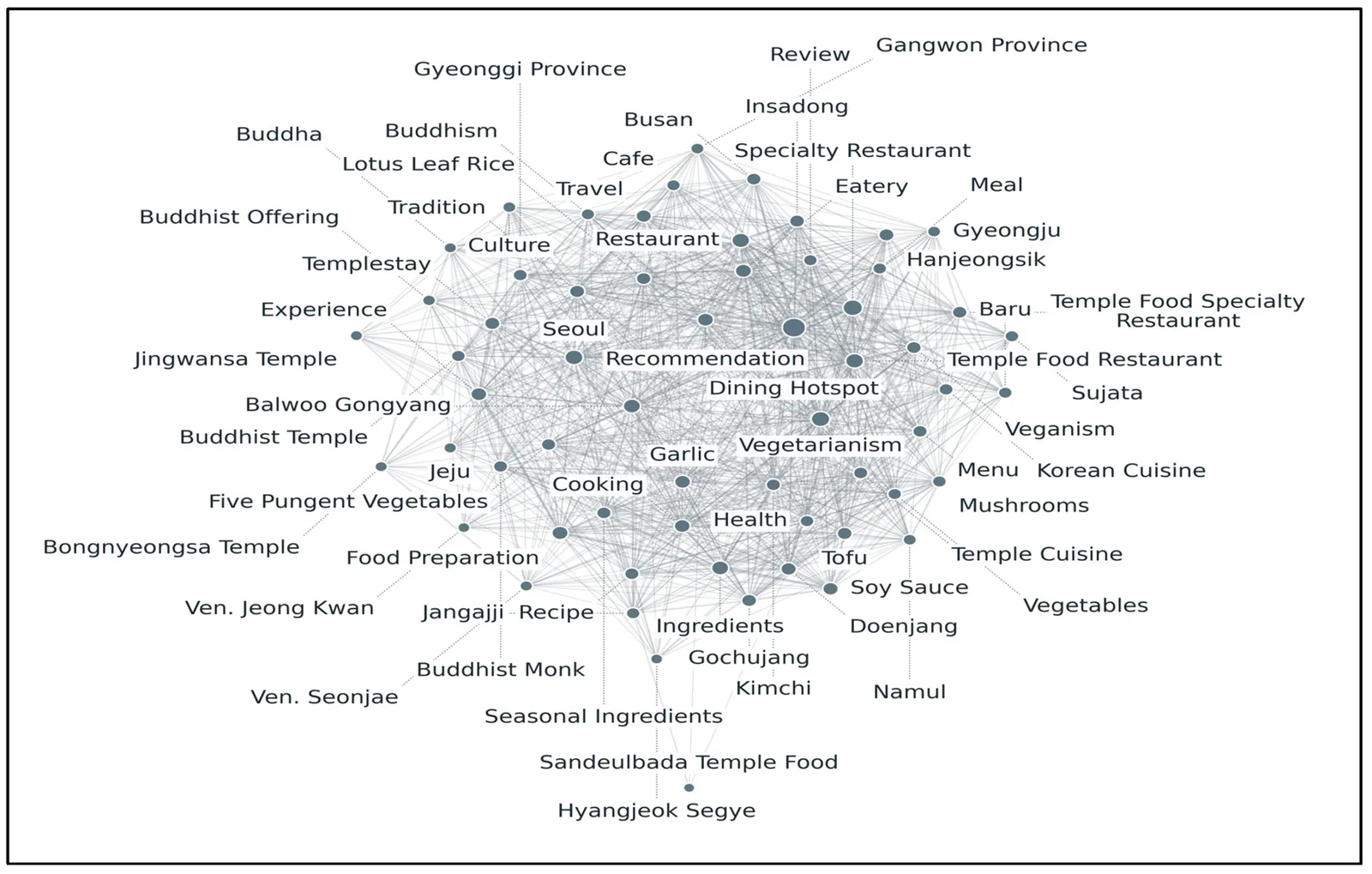
Full semantic network of Korean temple food keywords in the pre-COVID-19 period. All observed keyword co-occurrence ties are displayed. Node size represents normalized weighted strength, and edge width represents normalized co-occurrence intensity.

**Figure A2:**
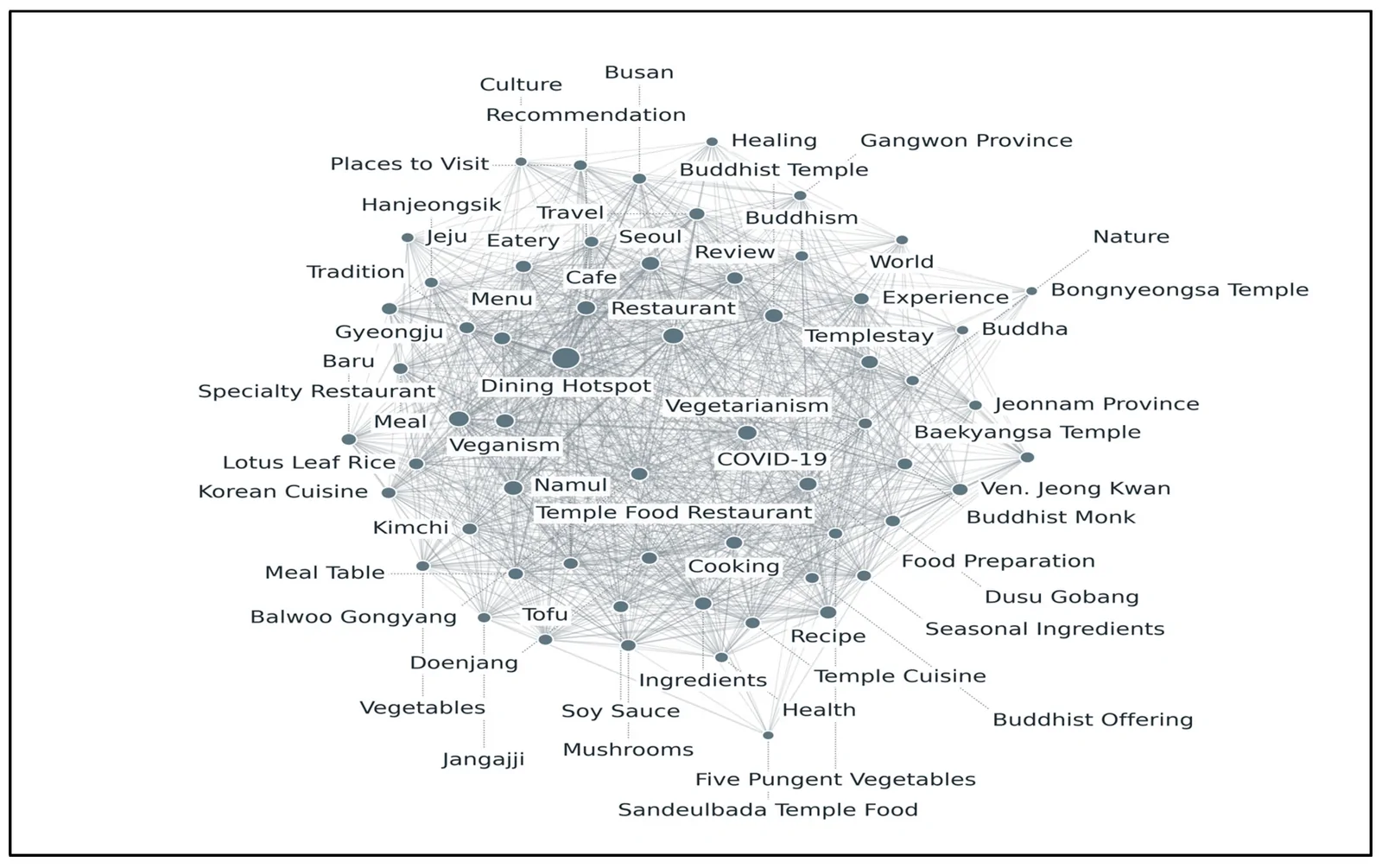
Full semantic network of Korean temple food keywords during the COVID-19 period. Node size indicates normalized weighted strength and edge width indicates normalized co-occurrence. All observed ties are shown.

**Figure A3:**
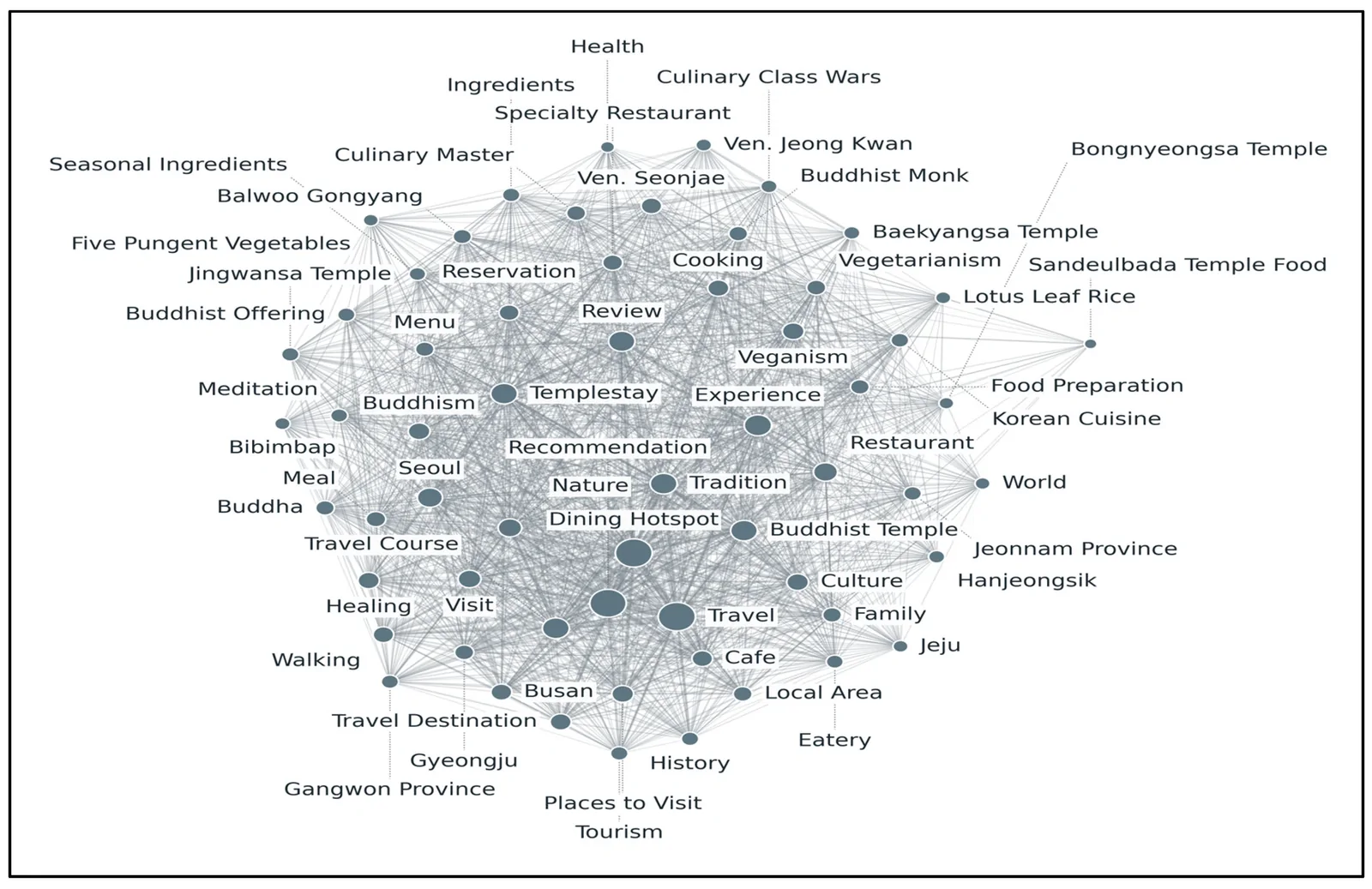
Full semantic network of Korean temple food keywords in the post-endemic period. Node size indicates normalized weighted strength and edge width indicates normalized co-occurrence. All observed ties are shown.

## Data Availability

The raw data are not publicly available due to platform terms of use and copyright restrictions governing online content. Processed data supporting the findings of this study are available from the corresponding author upon reasonable request.

## Appendix A

**Table A1:** Representative examples of data cleaning and keyword refinement.

Preprocessing Category	Representative Examples	Treatment Rule
Excluded documents	Duplicate posts; simple link collections; documents consisting mainly of symbols or numbers; irrelevant advertisements or promotional posts	Removed before analysis
Incidentally matched documents	Documents containing “사찰음식 (Korean temple food) ” but not substantively discussing temple food, Buddhist food culture, tourism, or related experiences	Removed as irrelevant
Stop-word and search-term examples	사찰음식 (Korean temple food), 사찰 (temple), 음식 (food), 한국 (Korea), 우리나라 (our country/Korea); particles, conjunctions, generic modifiers	Excluded from keyword analysis
Search-term handling	사찰음식 (Korean temple food), 사찰 (temple), 음식 (food)	Removed to reduce query bias
Merged synonyms and orthographic variants	템플스테이/템플 스테이 (Templestay); 발우공양/발우 공양 (Balwoo Gongyang); 연잎밥/연잎 밥 (lotus leaf rice); 코로나19 (COVID-19)/COVID-19	Merged into representative terms
Compound noun standardization	사찰음식점 (temple food restaurant), 사찰음식전문점 (temple food specialty restaurant), 템플스테이 (Templestay), 발우공양 (Balwoo Gongyang), 연잎밥 (lotus leaf rice), 제철재료 (seasonal ingredients), 오신채 (five pungent vegetables)	Standardized as single terms
Retained proper nouns and culturally specific expressions	선재스님 (Ven. Seonjae), 정관스님 (Ven. Jeong Kwan), 진관사 (Jingwansa Temple), 봉녕사 (Bongnyeongsa Temple), 백양사 (Baekyangsa Temple), 산들바다사찰음식 (Sandeulbada Temple Food), 흑백요리사 (Culinary Class Wars)	Retained when contextually meaningful
Retained food- and culture-specific terms	된장 (Doenjang), 간장 (soy sauce), 두부 (tofu), 나물 (Namul), 장아찌 (Jangajji), 오신채 (five pungent vegetables), 채식 (vegetarianism), 비건 (veganism), 명상 (meditation), 힐링 (healing)	Retained for semantic relevance
Removed foreign-language and noisy expressions	Irrelevant foreign terms, URLs, hashtags, product codes, fragmented expressions	Removed during refinement
Final keyword selection	Food, ingredients, Buddhist culture, vegetarian/vegan consumption, tourism, wellness, places, experts, and media-related terms	Selected 60 keywords per period

**Table A2:** Full list of 60 Korean temple food keywords and normalized frequencies by analytical period.

Rank	Period 1: Pre-COVID-19		Period 2: COVID-19		Period 3: Post-Endemic	
	Keyword	*n* (/1000)	Keyword	*n* (/1000)	Keyword	*n* (/1000)
1	Dining Hotspot	497 (63.5)	Dining Hotspot	651 (90.5)	Travel	1330 (142.5)
2	Seoul	241 (30.8)	Buddhist Temple	277 (38.5)	Dining Hotspot	1078 (115.5)
3	Vegetarianism	233 (29.8)	Seoul	243 (33.8)	Recommendation	912 (97.7)
4	Restaurant	219 (28.0)	Restaurant	243 (33.8)	Buddhist Temple	595 (63.7)
5	Food Preparation	217 (27.7)	Templestay	225 (31.3)	Templestay	572 (61.3)
6	Travel	206 (26.3)	Temple Food Restaurant	222 (30.9)	Buddhism	482 (51.6)
7	Specialty Restaurant	204 (26.1)	Food Preparation	219 (30.4)	Seoul	478 (51.2)
8	Busan	193 (24.7)	Vegetarianism	219 (30.4)	Experience	468 (50.1)
9	Ingredients	180 (23.0)	Buddhist Monk	216 (30.0)	Tradition	433 (46.4)
10	Eatery	179 (22.9)	Gyeongju	210 (29.2)	Busan	433 (46.4)
11	Buddhist Temple	171 (21.9)	Specialty Restaurant	202 (28.1)	Review	378 (40.5)
12	Experience	165 (21.1)	Veganism	200 (27.8)	Places to Visit	362 (38.8)
13	Temple Food Restaurant	165 (21.1)	Travel	196 (27.2)	Travel Course	362 (38.8)
14	Templestay	160 (20.5)	Recommendation	190 (26.4)	Ven. Seonjae	317 (34.0)
15	Cooking	159 (20.3)	Tradition	170 (23.6)	Restaurant	315 (33.7)
16	Balwoo Gongyang	159 (20.3)	Recipe	163 (22.7)	Gyeongju	315 (33.7)
17	Buddhist Monk	148 (18.9)	Baru	158 (22.0)	Nature	270 (28.9)
18	Recommendation	144 (18.4)	Busan	153 (21.3)	Veganism	266 (28.5)
19	Gyeonggi Province	142 (18.2)	Cooking	146 (20.3)	Visit	265 (28.4)
20	Tradition	140 (17.9)	Eatery	140 (19.5)	World	264 (28.3)
21	Veganism	139 (17.8)	Balwoo Gongyang	140 (19.5)	Buddhist Monk	258 (27.6)
22	Bongnyeongsa Temple	113 (14.4)	Gangwon Province	137 (19.0)	Culture	258 (27.6)
23	Temple Cuisine	111 (14.2)	Lotus Leaf Rice	136 (18.9)	Buddha	253 (27.1)
24	Garlic	111 (14.2)	Ingredients	129 (17.9)	Cooking	252 (27.0)
25	Recipe	109 (13.9)	Menu	125 (17.4)	Cafe	243 (26.0)
26	Gangwon Province	107 (13.7)	Buddhism	123 (17.1)	Food Preparation	234 (25.1)
27	Insadong	105 (13.4)	Dusu Gobang	119 (16.5)	Travel Destination	234 (25.1)
28	Jingwansa Temple	102 (13.0)	COVID-19	117 (16.3)	Gangwon Province	220 (23.6)
29	Buddhism	100 (12.8)	Jeonnam Province	115 (16.0)	Jingwansa Temple	203 (21.7)
30	Soy Sauce	98 (12.5)	Review	112 (15.6)	Bongnyeongsa Temple	201 (21.5)
31	Doenjang	96 (12.3)	Experience	108 (15.0)	Korean Cuisine	195 (20.9)
32	Lotus Leaf Rice	95 (12.1)	Cafe	107 (14.9)	Specialty Restaurant	193 (20.7)
33	Tofu	93 (11.9)	Ven. Jeong Kwan	105 (14.6)	Healing	181 (19.4)
34	Baru	93 (11.9)	Sandeulbada Temple Food	97 (13.5)	Culinary Master	179 (19.2)
35	Kimchi	92 (11.8)	Meal Table	96 (13.3)	Family	175 (18.7)
36	Sujata	92 (11.8)	Mushrooms	91 (12.6)	Vegetarianism	173 (18.5)
37	Ven. Seonjae	88 (11.2)	Jeju	91 (12.6)	Reservation	168 (18.0)
38	Temple Food Specialty Restaurant	88 (11.2)	Places to Visit	91 (12.6)	Local Area	166 (17.8)
39	Hanjeongsik	86 (11.0)	Jangajji	90 (12.5)	Meal	166 (17.8)
40	Health	84 (10.7)	Kimchi	88 (12.2)	Walking	163 (17.5)
41	Jangajji	84 (10.7)	Temple Cuisine	88 (12.2)	Ingredients	159 (17.0)
42	Hyangjeok Segye	84 (10.7)	Soy Sauce	84 (11.7)	Jeonnam Province	158 (16.9)
43	Cafe	83 (10.6)	Tofu	84 (11.7)	Balwoo Gongyang	146 (15.6)
44	Menu	82 (10.5)	Meal	83 (11.5)	Jeju	141 (15.1)
45	Jeju	78 (10.0)	Doenjang	81 (11.3)	Tourism	135 (14.5)
46	Review	78 (10.0)	Bongnyeongsa Temple	81 (11.3)	Eatery	132 (14.1)
47	Gyeongju	78 (10.0)	Namul	81 (11.3)	History	129 (13.8)
48	Gochujang	76 (9.7)	World	79 (11.0)	Menu	126 (13.5)
49	Seasonal Ingredients	75 (9.6)	Buddha	76 (10.6)	Buddhist Offering	123 (13.2)
50	Korean Cuisine	74 (9.5)	Baekyangsa Temple	73 (10.1)	Ven. Jeong Kwan	120 (12.9)
51	Culture	73 (9.3)	Seasonal Ingredients	71 (9.9)	Seasonal Ingredients	115 (12.3)
52	Buddha	73 (9.3)	Nature	70 (9.7)	Baekyangsa Temple	112 (12.0)
53	Buddhist Offering	64 (8.2)	Korean Cuisine	69 (9.6)	Lotus Leaf Rice	110 (11.8)
54	Ven. Jeong Kwan	63 (8.1)	Health	68 (9.4)	Culinary Class Wars	104 (11.1)
55	Namul	60 (7.7)	Buddhist Offering	65 (9.0)	Hanjeongsik	92 (9.9)
56	Meal	60 (7.7)	Vegetables	65 (9.0)	Bibimbap	87 (9.3)
57	Mushrooms	59 (7.5)	Five Pungent Vegetables	62 (8.6)	Meditation	85 (9.1)
58	Five Pungent Vegetables	59 (7.5)	Hanjeongsik	60 (8.3)	Five Pungent Vegetables	84 (9.0)
59	Vegetables	53 (6.8)	Culture	54 (7.5)	Health	76 (8.1)
60	Sandeulbada Temple Food	19 (2.4)	Healing	43 (6.0)	Sandeulbada Temple Food	52 (5.6)

Note. Keyword labels are presented in English for readability and cross-period comparability; they correspond to the standardized Korean terms used in the original text analysis. Representative Korean terms and normalization examples are provided in Appendix Table A1.

**Table A3:** Full network measures for 60 Korean temple food keywords by analytical period. Panel (**A**). Pre-COVID-19 period. Panel (**B**). COVID-19 period. Panel (**C**). Post-endemic period.

(**A**)				
**Frequency Rank**	**Keyword**	**NWS**	**DC**	**CC**
1	Dining Hotspot	60.335	0.915	0.922
2	Seoul	29.656	0.881	0.894
3	Vegetarianism	32.341	0.864	0.881
4	Restaurant	27.867	0.729	0.787
5	Food Preparation	20.58	0.695	0.766
6	Travel	16.873	0.627	0.728
7	Specialty Restaurant	37.198	0.797	0.831
8	Busan	13.422	0.559	0.694
9	Ingredients	25.182	0.661	0.747
10	Eatery	20.964	0.746	0.797
11	Buddhist Temple	10.738	0.576	0.702
12	Experience	19.43	0.661	0.747
13	Temple Food Restaurant	28.378	0.814	0.843
14	Templestay	16.49	0.627	0.728
15	Cooking	19.302	0.797	0.831
16	Balwoo Gongyang	25.054	0.678	0.756
17	Buddhist Monk	11.888	0.644	0.738
18	Recommendation	19.302	0.763	0.808
19	Gyeonggi Province	11.888	0.542	0.686
20	Tradition	16.234	0.695	0.766
21	Veganism	14.061	0.61	0.72
22	Bongnyeongsa Temple	4.73	0.288	0.584
23	Temple Cuisine	12.783	0.712	0.776
24	Garlic	19.43	0.814	0.843
25	Recipe	12.783	0.61	0.72
26	Gangwon Province	8.309	0.458	0.648
27	Insadong	15.212	0.576	0.702
28	Jingwansa Temple	4.73	0.254	0.567
29	Buddhism	9.204	0.593	0.711
30	Soy Sauce	17.257	0.559	0.694
31	Doenjang	17.001	0.695	0.766
32	Lotus Leaf Rice	13.933	0.746	0.797
33	Tofu	15.084	0.661	0.747
34	Baru	9.971	0.356	0.608
35	Kimchi	12.783	0.661	0.747
36	Sujata	10.226	0.373	0.615
37	Ven. Seonjae	5.241	0.441	0.641
38	Temple Food Specialty Restaurant	13.55	0.508	0.67
39	Hanjeongsik	15.723	0.542	0.686
40	Health	10.738	0.593	0.711
41	Jangajji	9.843	0.458	0.648
42	Hyangjeok Segye	4.091	0.271	0.578
43	Cafe	9.715	0.542	0.686
44	Menu	12.911	0.61	0.72
45	Jeju	5.624	0.407	0.628
46	Review	9.971	0.627	0.728
47	Gyeongju	7.798	0.441	0.641
48	Gochujang	15.212	0.542	0.686
49	Seasonal Ingredients	12.911	0.695	0.766
50	Korean Cuisine	11.632	0.627	0.728
51	Culture	7.542	0.424	0.634
52	Buddha	4.985	0.458	0.648
53	Buddhist Offering	6.903	0.424	0.628
54	Ven. Jeong Kwan	3.963	0.322	0.59
55	Namul	8.309	0.508	0.67
56	Meal	10.098	0.61	0.72
57	Mushrooms	10.482	0.61	0.72
58	Five Pungent Vegetables	12.655	0.593	0.702
59	Vegetables	9.843	0.644	0.738
60	Sandeulbada Temple Food	0.639	0.051	0.496
(**B**)				
**Frequency Rank**	**Keyword**	**NWS**	**DC**	**CC**
1	Dining Hotspot	99.778	0.898	0.908
2	Buddhist Temple	34.742	0.864	0.881
3	Seoul	33.769	0.797	0.831
4	Restaurant	49.055	0.898	0.908
5	Templestay	29.183	0.763	0.808
6	Temple Food Restaurant	38.494	0.763	0.808
7	Food Preparation	31.684	0.746	0.797
8	Vegetarianism	39.605	0.864	0.881
9	Buddhist Monk	19.038	0.729	0.787
10	Gyeongju	21.54	0.542	0.686
11	Specialty Restaurant	47.11	0.831	0.855
12	Veganism	34.603	0.763	0.808
13	Travel	21.818	0.644	0.738
14	Recommendation	35.019	0.864	0.881
15	Tradition	20.15	0.797	0.831
16	Recipe	27.515	0.627	0.728
17	Baru	18.066	0.525	0.678
18	Busan	14.036	0.508	0.67
19	Cooking	26.265	0.763	0.808
20	Eatery	22.374	0.678	0.756
21	Balwoo Gongyang	27.376	0.78	0.819
22	Gangwon Province	9.45	0.508	0.67
23	Lotus Leaf Rice	18.344	0.627	0.728
24	Ingredients	28.766	0.695	0.766
25	Menu	26.404	0.78	0.819
26	Buddhism	11.395	0.627	0.728
27	Dusu Gobang	18.066	0.576	0.702
28	COVID-19	13.619	0.763	0.808
29	Jeonnam Province	10.561	0.492	0.663
30	Review	24.18	0.797	0.831
31	Experience	19.872	0.814	0.843
32	Cafe	14.591	0.729	0.787
33	Ven. Jeong Kwan	21.401	0.559	0.694
34	Sandeulbada Temple Food	2.918	0.186	0.551
35	Meal Table	19.594	0.712	0.776
36	Mushrooms	18.899	0.61	0.72
37	Jeju	8.06	0.424	0.634
38	Places to Visit	12.09	0.441	0.641
39	Jangajji	10.422	0.627	0.728
40	Kimchi	18.482	0.729	0.787
41	Temple Cuisine	17.788	0.678	0.756
42	Soy Sauce	20.15	0.695	0.766
43	Tofu	14.73	0.593	0.711
44	Meal	17.927	0.627	0.728
45	Doenjang	22.513	0.814	0.843
46	Bongnyeongsa Temple	3.474	0.288	0.584
47	Namul	17.788	0.797	0.831
48	World	7.365	0.441	0.641
49	Buddha	5.837	0.458	0.648
50	Baekyangsa Temple	14.314	0.458	0.648
51	Seasonal Ingredients	16.12	0.661	0.747
52	Nature	10.145	0.661	0.747
53	Korean Cuisine	16.12	0.627	0.728
54	Health	11.117	0.576	0.702
55	Buddhist Offering	13.758	0.644	0.738
56	Vegetables	12.09	0.593	0.711
57	Five Pungent Vegetables	13.897	0.695	0.766
58	Hanjeongsik	12.09	0.542	0.686
59	Culture	5.003	0.373	0.615
60	Healing	5.698	0.39	0.621
(**C**)				
**Frequency Rank**	**Keyword**	**NWS**	**DC**	**CC**
1	Travel	177.844	0.966	0.967
2	Dining Hotspot	174.309	0.983	0.983
3	Recommendation	169.809	0.966	0.967
4	Buddhist Temple	83.351	0.949	0.952
5	Templestay	83.458	0.949	0.952
6	Buddhism	49.496	0.949	0.952
7	Seoul	71.888	0.932	0.937
8	Experience	87.958	1	1
9	Tradition	84.53	1	1
10	Busan	44.033	0.746	0.797
11	Review	80.673	0.966	0.967
12	Places to Visit	49.496	0.797	0.831
13	Travel Course	85.172	0.949	0.952
14	Ven. Seonjae	42.104	0.797	0.831
15	Restaurant	64.174	0.949	0.952
16	Gyeongju	34.176	0.797	0.831
17	Nature	60.531	1	1
18	Veganism	49.925	0.949	0.952
19	Visit	56.139	0.983	0.983
20	World	14.249	0.712	0.776
21	Buddhist Monk	32.676	0.831	0.855
22	Culture	49.282	0.898	0.908
23	Buddha	31.498	0.847	0.868
24	Cooking	45.961	0.932	0.937
25	Cafe	42.426	0.881	0.894
26	Food Preparation	32.462	0.881	0.894
27	Travel Destination	45.747	0.746	0.797
28	Gangwon Province	24.534	0.678	0.756
29	Jingwansa Temple	25.927	0.678	0.756
30	Bongnyeongsa Temple	13.178	0.695	0.766
31	Korean Cuisine	27.748	0.712	0.776
32	Specialty Restaurant	38.997	0.78	0.819
33	Healing	47.032	0.814	0.843
34	Culinary Master	34.176	0.814	0.843
35	Family	32.891	0.847	0.868
36	Vegetarianism	34.498	0.881	0.894
37	Reservation	39.961	0.966	0.967
38	Local Area	33.319	0.763	0.808
39	Meal	37.39	0.932	0.937
40	Walking	42.533	0.763	0.808
41	Ingredients	26.248	0.814	0.843
42	Jeonnam Province	26.248	0.831	0.855
43	Balwoo Gongyang	30.534	0.729	0.787
44	Jeju	14.785	0.746	0.797
45	Tourism	25.712	0.678	0.756
46	Eatery	23.355	0.78	0.819
47	History	25.498	0.678	0.756
48	Menu	31.283	0.881	0.894
49	Buddhist Offering	25.498	0.78	0.819
50	Ven. Jeong Kwan	17.249	0.627	0.728
51	Seasonal Ingredients	21.856	0.847	0.868
52	Baekyangsa Temple	20.034	0.695	0.766
53	Lotus Leaf Rice	15.535	0.678	0.756
54	Culinary Class Wars	20.463	0.763	0.808
55	Hanjeongsik	19.499	0.661	0.747
56	Bibimbap	16.285	0.746	0.797
57	Meditation	23.784	0.864	0.881
58	Five Pungent Vegetables	14.463	0.678	0.756
59	Health	10.606	0.644	0.738
60	Sandeulbada Temple Food	5.25	0.288	0.584

**Table A4:** Jaccard similarity sensitivity across alternative keyword cutoffs.

Keyword Cutoff	Pre-COVID-19 vs. COVID-19	COVID-19 vs. Post-Endemic	Pre-COVID-19 vs. Post-Endemic
Top 40	0.481	0.379	0.356
Top 60	0.739	0.6	0.519
Top 100	0.695	0.587	0.575

Note: Jaccard coefficients varied by cutoff, but the relative ordering of period comparisons remained unchanged.

**Table A5:** Document-level bootstrap robustness of network measures. Panel (**A**). Stability of NWS Rankings. Panel (**B**). Bootstrap intervals for network-level differences.

(**A**)		
**Period**	**Median Spearman ρ**	**95% Bootstrap Interval**
Pre-COVID-19	0.953	0.923–0.972
COVID-19	0.961	0.935–0.979
Post-Endemic	0.981	0.971–0.989
(**B**)		
**Comparison**	**Density Difference**	**Average Shortest-Path Length Difference**
	**95% Interval**	**95% Interval**
Pre-COVID-19 to COVID-19	0.038 to 0.094	−0.112 to −0.042
COVID-19 to Post-Endemic	0.168 to 0.219	−0.219 to −0.169
Pre-COVID-19 to Post-Endemic	0.236 to 0.286	−0.303 to −0.241

Note: Results are based on 1000 document-level bootstrap resamples within each period.

**Table A6:** Modularity-based robustness of the CONCOR cluster structure.

Period	Louvain Results	Greedy Modularity	Interpretation
Pre-COVID-19	K = 4 in 69/100 runs	Q4 = 0.2173; Q5 = 0.2174	Four-cluster solution near-optimal
COVID-19	K = 4 in 100/100 runs	Q4 = 0.2305 (highest)	Strong support for four clusters
Post-Endemic	K = 3 in 97/100 runs; K = 4 in 3/100	Q3 = 0.1792; Q4 = 0.1783	Three-community macrostructure slightly preferred

Note: Louvain analyses used 100 random starts and greedy modularity examined two to eight communities. CONCOR and modularity were interpreted as complementary rather than equivalent classifications.
